# The secretome of the fish pathogen *Tenacibaculum maritimum* includes soluble virulence-related proteins and outer membrane vesicles

**DOI:** 10.3389/fcimb.2023.1197290

**Published:** 2023-06-09

**Authors:** M. Pilar Escribano, Miguel Balado, Alicia E. Toranzo, Manuel L. Lemos, Beatriz Magariños

**Affiliations:** Department of Microbiology and Parasitology, Institute of Aquaculture and Faculty of Biology-CIBUS, University of Santiago de Compostela, Santiago de Compostela, Spain

**Keywords:** *Tenacibaculum maritimum*, outer membrane vesicles (OMVs), virulence factors, tenacibaculosis, extracellular products (ECPs), hydrolytic enzymatic activities

## Abstract

*Tenacibaculum maritimum*, the etiological agent of tenacibaculosis in marine fish, constitutively secretes extracellular products (ECPs) in which protein content has not been yet comprehensively studied. In this work, the prevalence of extracellular proteolytic and lipolytic activities related to virulence was analyzed in 64 *T. maritimum* strains belonging to the O1–O4 serotypes. The results showed the existence of a great intra-specific heterogeneity in the enzymatic capacity, particularly within serotype O4. Thus, the secretome of a strain belonging to this serotype was characterized by analyzing the protein content of ECPs and the possible production of outer membrane vesicles (OMVs). Notably, the ECPs of *T. maritimum* SP9.1 contain a large amount of OMVs that were characterized by electron microscopy and purified. Thus, ECPs were divided into soluble (S-ECPs) and insoluble fractions (OMVs), and their protein content was analyzed by a high-throughput proteomic approach. A total of 641 proteins were identified in ECPs including some virulence-related factors, which were mainly found in one of the fractions, either OMVs or S-ECPs. Outer membrane proteins such as TonB-dependent siderophore transporters and the type IX secretion system (T9SS)-related proteins PorP, PorT, and SprA appeared to be mainly associated with OMVs. By contrast, putative virulence factors such as sialidase SiaA, chondroitinase CslA, sphingomyelinase Sph, ceramidase Cer, and collagenase Col were found only in the S-ECPs. These findings clearly demonstrate that *T. maritimum* releases, through surface blebbing, OMVs specifically enriched in TonB-dependent transporters and T9SS proteins. Interestingly, *in vitro* and *in vivo* assays also showed that OMVs could play a key role in virulence by promoting surface adhesion and biofilm formation and maximizing the cytotoxic effects of the ECPs. The characterization of *T. maritimum* secretome provides insights into ECP function and can constitute the basis for future studies aimed to elucidate the full role of OMVs in the pathogenesis of fish tenacibaculosis.

## Introduction

The genus *Tenacibaculum* includes Gram-negative bacteria, which are aerobic, filamentous, and motile by gliding, belonging to the family *Flavobacteriaceae* (Wakabayashi et al., 1986; Suzuki et al., 2001; Kim et al., 2017; Park et al., 2018). Several *Tenacibaculum* species are identified as potential fish pathogens worldwide (Fernández-Álvarez and Santos, 2018). *Tenacibaculum maritimum* is the main etiological agent of tenacibaculosis, a marine pathology associated with gross lesions on the fish body (Avendaño-Herrera et al., 2005; Toranzo et al., 2005). These skin lesions typically show erosions containing many long filamentous bacteria forming adherent mats over the eroded surface that extend deeply into the connective tissue layer (Handlinger et al., 1997; Frisch et al., 2018; Mabrok et al., 2023).

The complete genome sequence of *T. maritimum* provided insights into concrete virulence-associated genes encoding the biosynthesis of exopolysaccharides, a type IX secretion system (T9SS), iron uptake systems, adhesins, hemolysins, proteases, and glycoside hydrolases (Pérez-Pascual et al., 2017). However, despite the significance of tenacibaculosis outbreaks in the aquaculture industry, knowledge about the molecular basis of *T. maritimum* pathogenesis is scarce. Four different lipopolysaccharide (LPS) serotypes and eight sub-groups have been described in *T. maritimum* based on the O antigen (Avendaño-Herrera et al., 2005; López et al., 2022). Adhesion to biotic and abiotic surfaces, gliding motility, hemagglutination, and production of extracellular products (ECPs) have been suggested to play major roles in *T. maritimum* pathogenesis (Baxa et al., 1988; Pazos, 1997; van Gelderen et al., 2009; Escribano et al., 2020; Mabrok et al., 2023). *T. maritimum* strongly attaches to different substrata including the skin and mucus of fish and tends to develop profuse biofilms, properties that enhance environmental persistence and tenacibaculosis transmission over time (Magariños et al., 1995; Levipan et al., 2019). The newly identified T9SS has been suggested as the main mechanism of virulence since it likely would aid the colonization of fish tissues by bacteria of the *Bacteroidetes* phylum (McBride, 2019). T9SS enables the gliding motility of bacteria, enhances adhesion to biotic and abiotic surfaces, and helps in biofilm formation (Eckroat et al., 2021). It also mediates the transport of some virulence factors to the cell surface or extracellular space (McBride, 2001; McBride and Zhu, 2013; Veith et al., 2015). In fact, non-gliding *T. maritimum* isolates were found avirulent in fish immersion challenges, showing lower adhesion ability to the glass wall and the skin fish surface (Rahman et al., 2014).

*T. maritimum* constitutively secretes ECPs that display numerous lytic activities such as gelatinase, caseinase, amylase, or hemolysis (Baxa et al., 1988; Pazos, 1997; van Gelderen et al., 2009). It is known that ECPs play a main role in *T. maritimum* virulence, but they have not been comprehensively analyzed so far. In addition, *T. maritimum* ECPs contain high levels of LPS (van Gelderen et al., 2010), which would suggest the possible release of some types of membrane vesicles (outer membrane vesicles (OMVs)) from the bacterial cells. In this concern, the production of OMVs was described in some Gram-negative bacteria (Ávila-Calderón et al., 2021) including fish pathogens such as *Photobacterium damselae* subsp. *piscicida*, *Vibrio ordalii*, *Vibrio anguillarum*, *Vibrio fischeri*, *Piscirickettsia salmonis*, *Flavobacterium columnare*, or *Flavobacterium psycrophyllum* (Møller et al., 2005; Hong et al., 2009; Laanto et al., 2014; Aschtgen et al., 2016; Oliver et al., 2016; Echevarría-Bugueño et al., 2020; Teixeira et al., 2023). The present work aims to characterize the secretome of *T. maritimum*, analyzing the extracellular proteolytic and lipolytic activities related to virulence and the possible production of OMVs. ECPs were divided into soluble (S-ECPs) and insoluble fractions (OMVs), and their protein content was identified using a high-throughput proteomic approach. Functional analysis showed that both fractions, OMVs and S-ECPs, are needed to achieve maximal levels of extracellular enzymatic activities, biofilm formation, and toxicity. These findings showed that *T. maritimum* release, through surface blebbing, outer membrane vesicles that would play a major role in virulence by promoting surface adhesion and biofilm formation and maximizing the cytotoxic effects of the ECPs. The secretome profile described here provides insights into the extracellular product function and constitutes a basis for future studies to elucidate the role of OMVs in tenacibaculosis pathogenesis.

## Results

### Prevalence of virulence-related enzymatic activities in a collection of *T. maritimum* strains belonging to serotypes O1–O4

To study the prevalence of virulence-related enzymatic activities in *T. maritimum*, a collection of 64 virulent strains representing the four main serotypes was screened for lysis of erythrocytes and some hydrolytic activities related to virulence. The results obtained are summarized in **Table 1**. All strains showed weak α-hemolysis, producing a discolored hemolytic halo between 2 and 5 mm wide on sheep blood agar plates. By contrast, none of the strains tested showed elastase activity. All *T. maritimum* isolates of serotypes O1, O2, and O3 showed almost the same phenotype, being positive for esterase, lipase, phospholipase, gelatinase, caseinase, and chondroitinase activities. However, up to four different hydrolytic activity profiles were found among O4 strains (**Table 1**; **Supplementary Figure S2**). Thus, only caseinase and chondroitinase activities were detected in all *T. maritimum* isolates (**Table 1**). These results indicate the existence of some grade of phenotypic heterogeneity among the *T. maritimum* serotype O4 isolates. According to these results, *T. maritimum* strain SP9.1 was selected for further analysis since it belongs to serotype O4 and shows positive hydrolytic activities for all the assayed substrates (except elastin).

**Table 1:** Hydrolytic activity profiles of a collection of 64 *Tenacibaculum maritimum* strains belonging to serotypes O1, O2, O3, and O4.

Serotype	Lipolytic activities			Proteolytic activities			Polysaccharide hydrolysis	Hemolytic activities
	Esterase	Lipase	Phospholipase	Gelatinase	Caseinase	Elastase	Chondroitinase	α-Hemolysis
O1 (n = 16)	+	+	+	+	+	−	+	+
O2 (n = 16)	+	+	+	+	+	−	+	+
O3 (n = 16)	+	+	+	+	+	−	+	+
O4 (n = 6)	+	+	+	+	+	−	+	+
O4 (n = 5)	−	+	−	+	+	−	+	+
O4 (n = 4)	±	±	−	+	+	−	+	+
O4 (n = 1)	−	−	−	−	−	−	+	+

Sixteen strains of each serotype were tested. Hydrolytic activities are indicated as the presence (+), absence (−), or weak (±) activity in tested isolates. The value of “n” indicates the number of strains that present each profile.

### *T. maritimum* releases outer membrane vesicles

**Figure 1:**
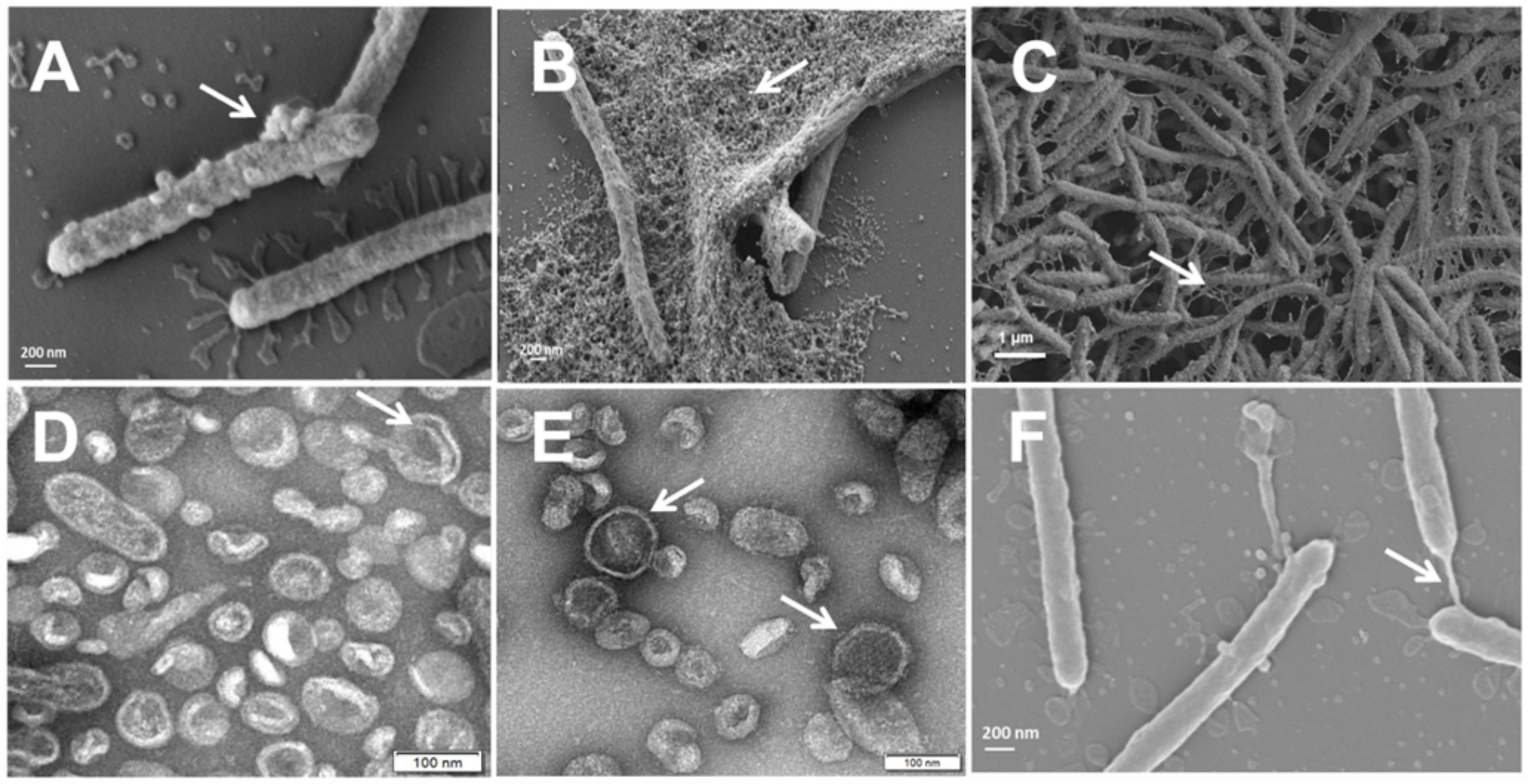
*Tenacibaculum maritimum* cells observed by scanning electron microscopy (SEM) **(A–C, F)** and outer membrane vesicle (OMV) fraction negatively stained with 2% uranyl acetate observed by transmission electron microscopy (TEM) **(D, E)**. *T. maritimum* surface blebs and biofilm production after 10 h **(A)** and at 24 h **(B, C)** of incubation. Isolated spheric OMVs of approximately 100 µm in diameter adhering to each other **(D, E)**. Surface coat structure (gliding) **(C, F)**.

*T. maritimum* SP9.1 biofilm formation and active attachment to glass coverslips were monitored by scanning electron microscopy (SEM) at 10 and 24 h of incubation. As shown in **Figure 1**, *T. maritimum* cells colonizing glass coverslips did not show pili or flagella structures. Surface blebbing and many small and large spherical structures that would be probable vesicles were observed at both times (**Figures 1A, B**). To test whether *T. maritimum* secretes extracellular vesicles, the ECPs of *T. maritimum* SP9.1 were isolated and fractioned into soluble (S-ECPs) and insoluble fractions since we hypothesized that the insoluble fraction of the ECPs would be enriched in OMVs. The insoluble fraction was also observed by transmission electron microscopy (TEM). Results showed that the insoluble fraction of the ECPs mainly consists in spherical structures that would be monolayered outer membrane vesicles (**Figures 1D, E**). A low proportion of these vesicles could have a bilayer membrane (**Figure 1D**). Notably, blebs and vesicles were close in size, ranging from 0.1 to 0.8 µm in length and up to 100 nm in diameter. During attachment, glass coverslips and bacterial surfaces were covered by the vesicles, forming aggregates with numerous round OMVs adhering to each other to finally form a dense matrix or biofilm (**Figures 1B, C**). An extracellular and adherent matrix with long blebs connecting some cells among them and to the coverslip was also observed from the first 10 h of colonization (**Figures 1B, C, F**). The proteome content characterization of the insoluble fraction (see below) showed that it consists mainly of OMVs. Thus, the insoluble fraction of the *T. maritimum* ECPs will henceforth be called OMV fraction.

### *T. maritimum* secretome characterization

**Figure 2:**
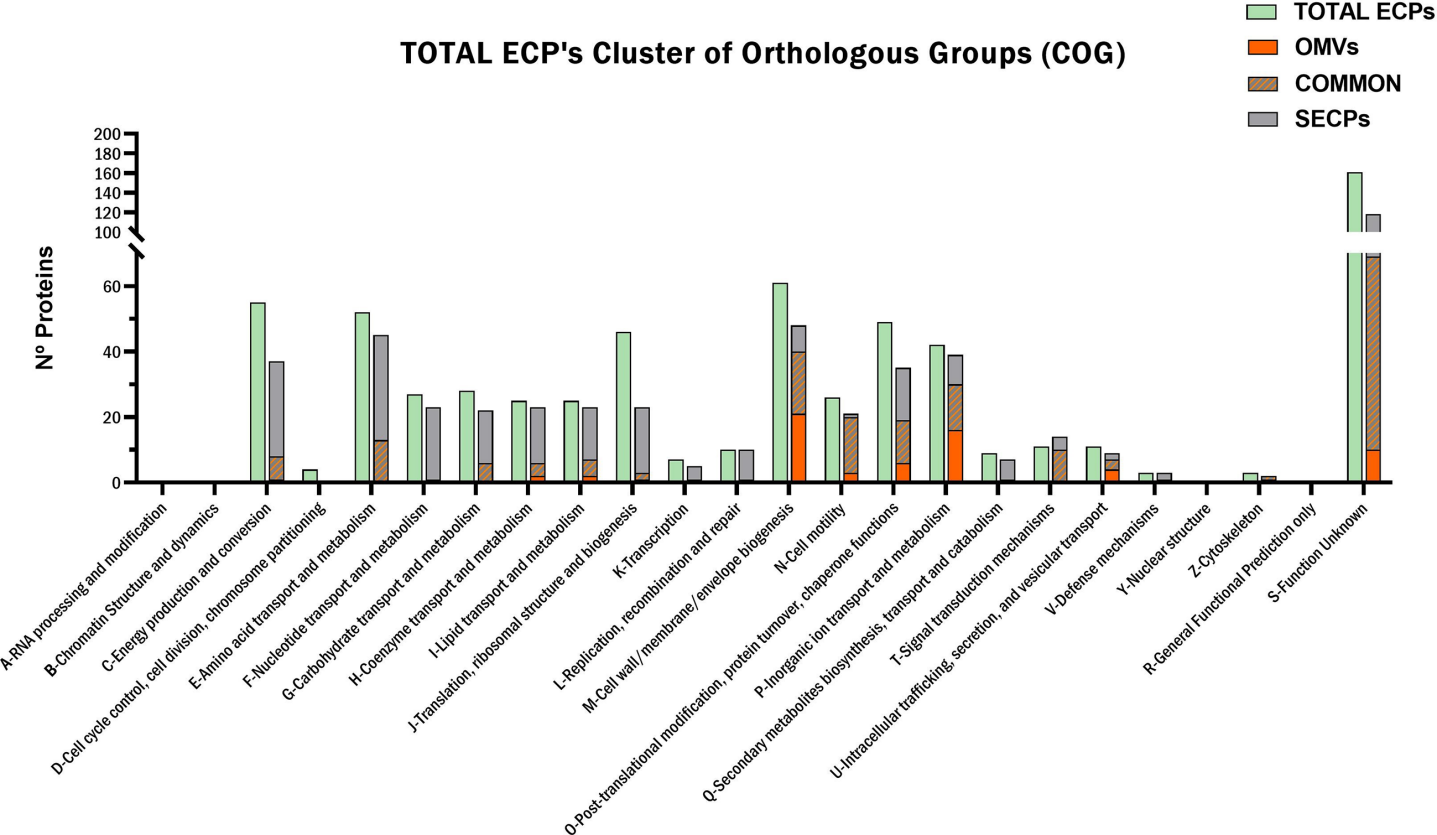
Functional KEGG classification of the *Tenacibaculum maritimum* ECP protein content. KEGG, Kyoto Encyclopedia of Genes and Genomes; ECP, extracellular product.

**Figure 3:**
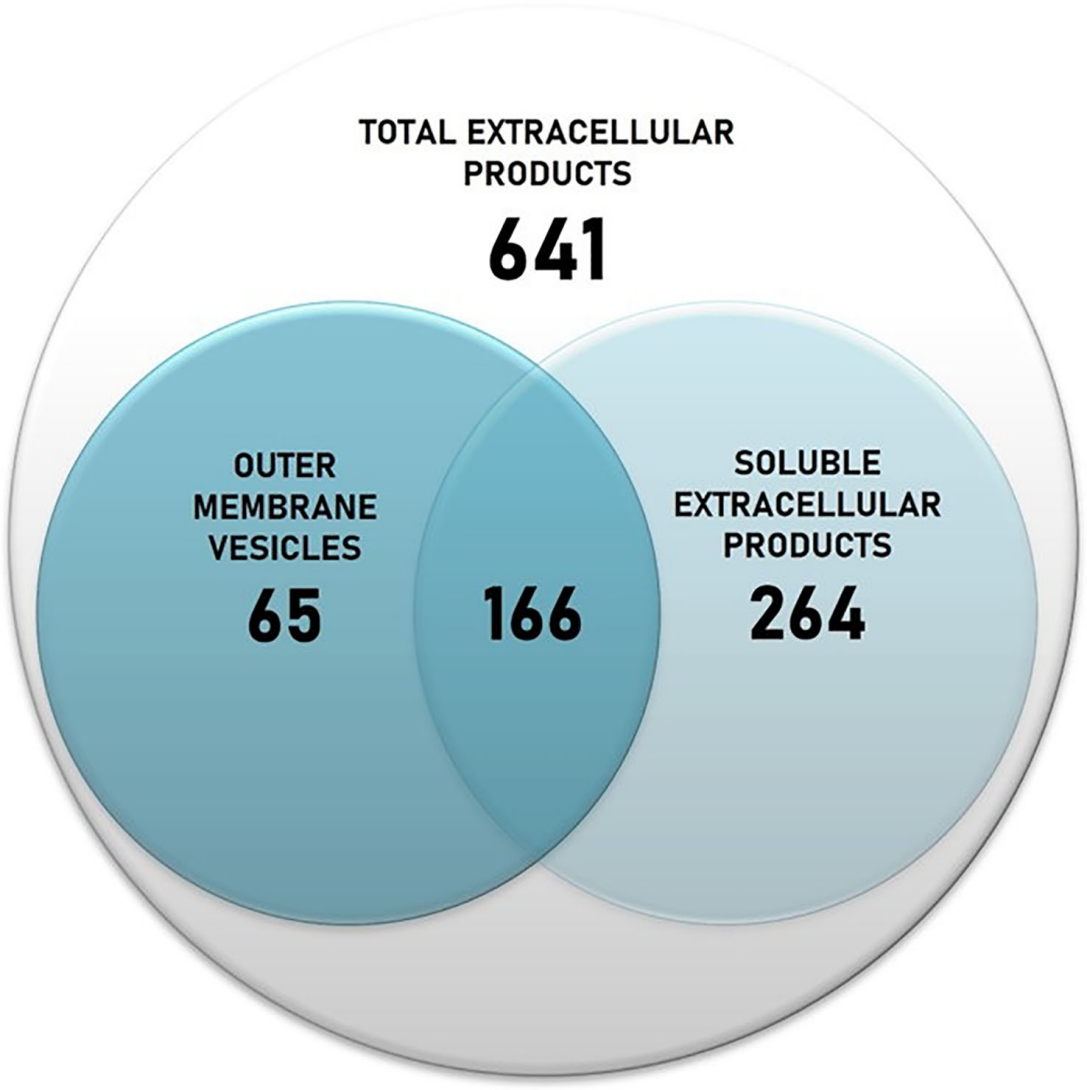
Number of proteins identified in the *Tenacibaculum maritimum* total ECPs and number of proteins recovered after fractionation in S-ECPs and OMVs. ECPs, extracellular products; S-ECPs, soluble extracellular products; OMVs, outer membrane vesicles.

The protein content of *T. maritimum* ECPs was determined using a high-throughput proteomic approach using a MSMS analysis in timsPro equipment, after tryptic digestion and a previous separation by reverse phase chromatography (nLC-TIMS-QTOF). Since iron starvation is one of the signals that pathogens found when they are colonizing the host, promoting the expression of most virulence factors (Cassat and Skaar, 2013), the ECPs were purified from *T. maritimum* SP9.1 cultures grown under low iron conditions (see Materials and Methods). A total of 641 non-redundant proteins were identified in the ECPs, which would represent ca. 22.36% of the theoretical proteome of *T. maritimum* SP9.1. For a better interpretation of the results, the proteins identified in cell-free ECPs or one of the fractions were classified into functional Kyoto Encyclopedia of Genes and Genomes (KEGG) categories (**Figure 2**). The most represented functional groups of the ECPs were those related to cell membrane biogenesis (M), energy production and conversion (C), amino acid metabolism and transport (E), post-translational modification (O), inorganic ion transport and metabolism (P), and cell motility (N). The proteins that were specifically associated with OMVs were classified almost exclusively into cell membrane biogenesis (M), cell motility (N), post-translational modification (O), and inorganic ion transport and metabolism (P). A total of 495 proteins of the 641 identified in the total ECPs were recovered and analyzed after the fractionation between S-ECPs and OMVs (**Figure 3**). Thus, all the proteins found in one of the fractions (S-ECPs or OMVs) were unambiguously identified as part of *T. maritimum* total ECPs (**Figure 3**). A total of 264 proteins were found exclusively in S-ECPs and 65 in OMVs, and 166 were common to both S-ECP and OMV fractions (**Figure 3**). Those proteins previously reported to be related to virulence are summarized in **Supplementary Table S2**.

**Figure 4:**
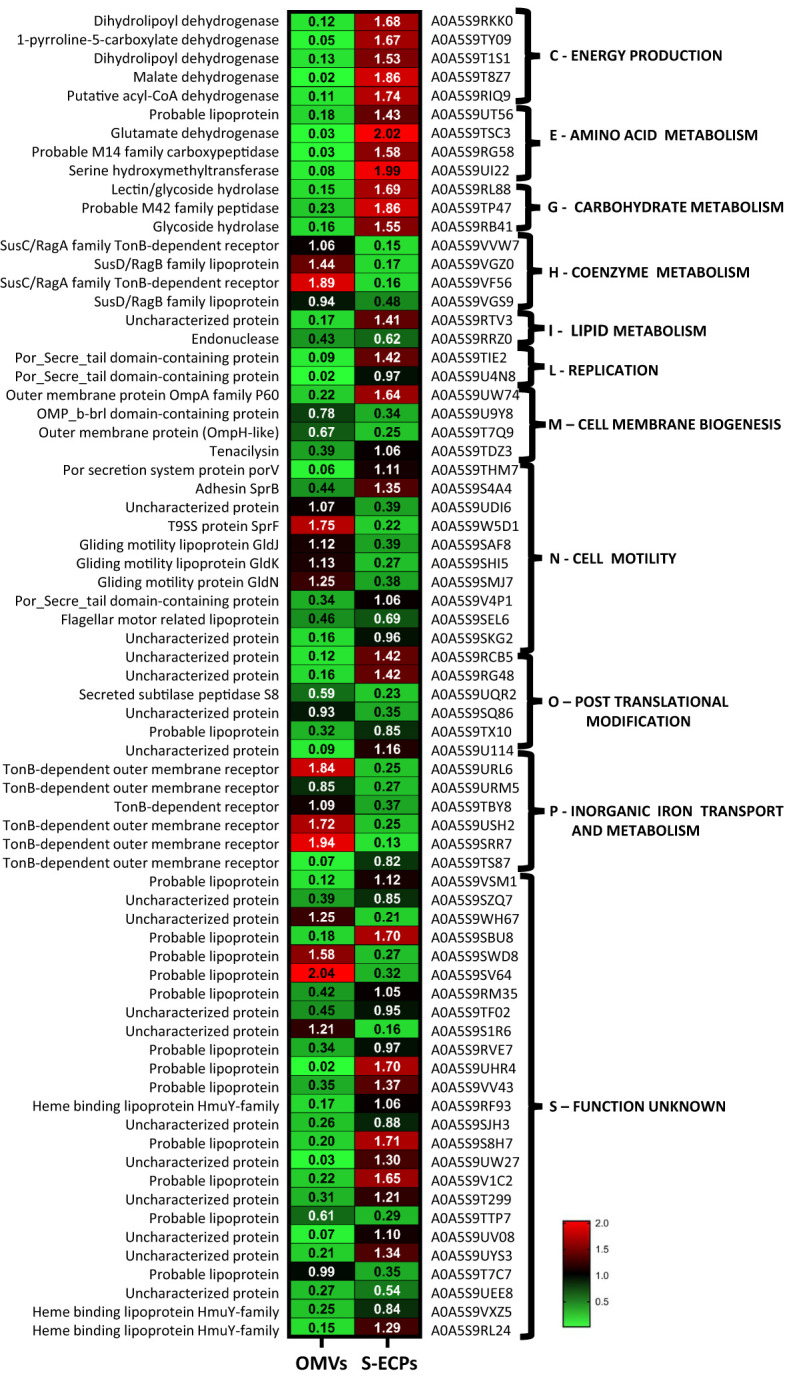
Proteins identified in both OMV and S-ECP fractions whose relative quantification showed statistically significant differences. A double criteria of *p*-values<0.05 and fold change cutoff ≥2 were used to assume the existence of statistical differences. OMV, outer membrane vesicle; S-ECP, soluble extracellular product.

**Figure 5:**
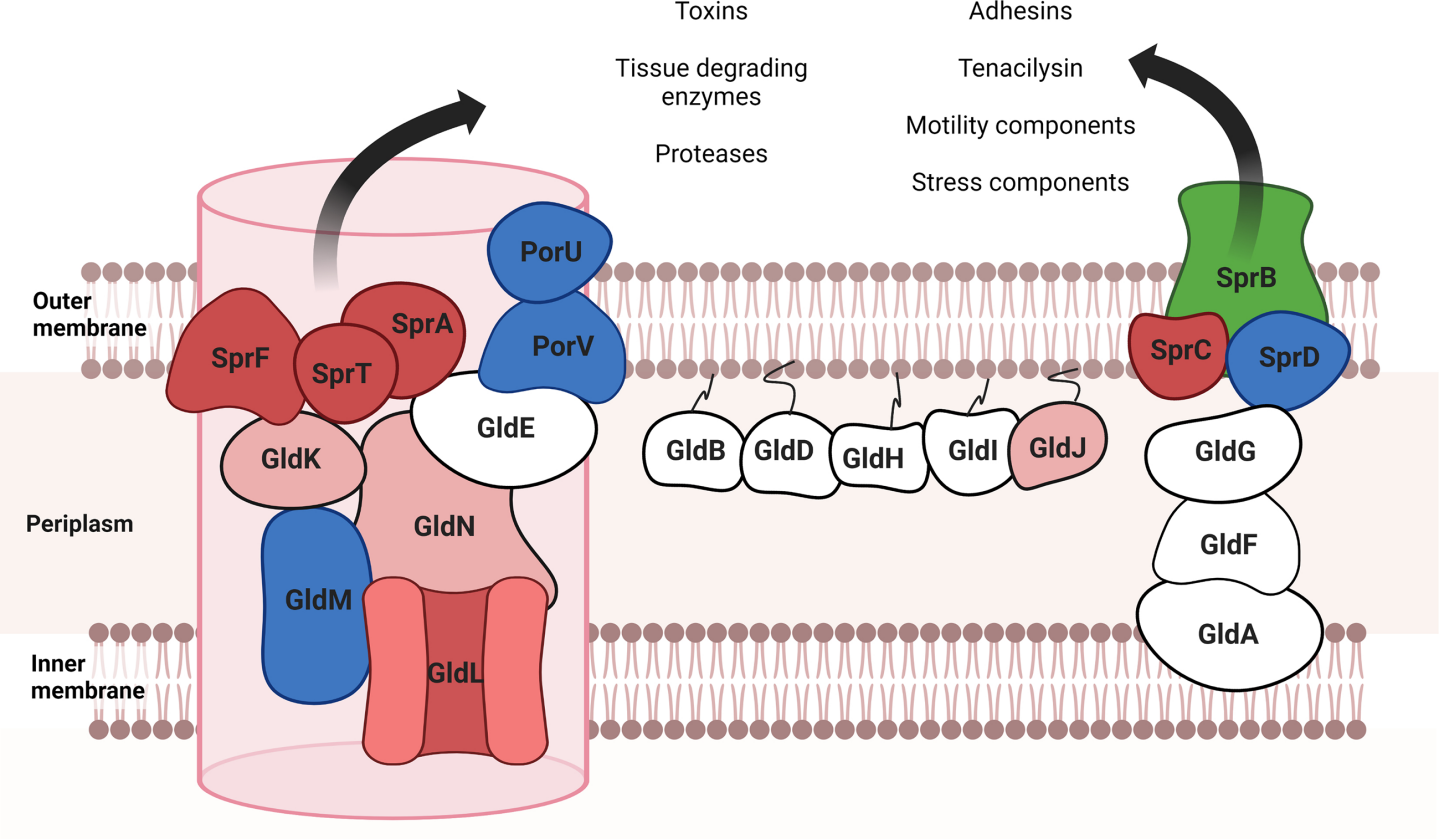
T9SS and motility proteins presented in extracellular products of *Tenacibaculum maritimum* (adapted from McBride and Nakane, 2015). In red are indicated the proteins that only appeared in the OMV fraction; in clear red, the proteins that were significantly more abundant in OMVs; in green, the proteins that appeared with a higher representation in the S-ECP fraction; in blue, the proteins that were detected in both fractions without significant differences. Proteins that did not appear in total ECPs are represented in white. Created with BioRender.com.

Presence/absence analysis (**Supplementary Table S3**) was performed, and the relative abundance of the proteins that appeared in both fractions was analyzed to infer their location in each ECP fraction (**Supplementary Table 4**; **Figure 4**). The most abundant proteins of the secretome were outer membrane-associated proteins such as components of the T9SS, TonB-dependent outer membrane transporters, and lipoproteins. Cytoplasmic proteins were not detected in ECPs, which indicates that possible contamination with whole cells or cell debris was below our detection limit. Most components of the gliding motility machinery and T9SS described in *Flavobacterium* (Gorasia et al., 2020) were identified as part of the *T. maritimum* secretome (**Supplementary Table S2**; **Figure 5**). Proteins PorU, PorV, GldM, and SprD were present at equal levels in both ECP fractions. However, proteins related to T9SS functions, such as GldK, GldJ, and GldN, were significantly in higher proportions in OMVs than in S-ECPs. In addition, proteins GldL, SprF, SprT, SprA, and SprC were only detected in the OMVs. Interestingly, adhesin SprB and another putative adhesin (A0A5S9SKG2_9FLAO) were threefold more abundant in S-ECPs than in OMVs. Most of the T9SS components associated with the inner membrane or periplasm (GldA, GldB, GldD, GldE, GldG, GldH, GldI, and GldF) were not detected (**Figure 5**). The absence of most of the inner membrane proteins confirms that the vesicles purified were primarily derived from the outer membrane (OM).

Cell membrane biogenesis (M) and inorganic ion transport and metabolism (P) were two of the functional KEGG categories with more representation in the extracellular products, with a total of 61 and 42 predicted proteins, respectively, being mostly of them outer membrane proteins (**Figure 2**). In fact, numerous lipoproteins and TonB-dependent outer membrane transporters (TBDTs) were found among the most abundant proteins of the *T. maritimum* ECPs (**Supplementary Table S3**). Most of the lipoproteins were present in higher amounts in the S-ECPs, while the TBDTs were found mainly associated with the OMVs (**Figure 4**; **Supplementary Table S2**). Seventeen different TBDTs putatively involved in iron uptake were detected in higher quantities in the OMV fraction (**Supplementary Table S2**). They include the TBDT (A0A5S9URL6) most likely involved in the internalization of the siderophore produced by *T. maritimum* since it is one of the most abundant in the ECPs, and it is encoded within a siderophore biosynthesis gene cluster. Interestingly, it should be noted that in this region of the genome, six more putative TBDTs (A0A5S9TN86, A0A5S9VD89, A0A5S9URM5, A0A5S9TCC5, A0A5S9TBY8, and A0A5S9TCA2) have been identified, being all of them mainly associated with OMVs (**Supplementary Table S2**). Two other major proteins identified in the *T. maritimum* secretome were the heme-binding lipoprotein HmuY (A0A5S9RL24) and the TBDT HmuR (A0A5S9RJX3), which was predicted to play a role in heme uptake in other *Bacteroidetes* species (Conrad et al., 2022; Olczak et al., 2008; Zhu et al., 2022). A homolog of the ferric citrate TBDT FecA (A0A5S9U4N9) and four associated lipoproteins annotated as “Imelysin-like proteins” (A0A5S9RU32, A0A5S9RVE7, A0A5S9RVP5, and A0A5S9U114) were also present in great abundance in the OMV fraction. Notably, among the most abundant proteins of the ECPs (**Supplementary Table S3**), two TBDT (A0A5S9VF56_9FLAO and A0A5S9VVW7) members of the SusC/RagA family and two surface lipoproteins (A0A5S9VGZ0 and A0A5S9VGS9) belonging to SusD superfamily were identified. These functions are present in higher amounts in OMVs and, altogether, would form the so-called “SusC/SusD homolog pair” that were postulated to mediate glycan harvesting from host glycoproteins (Pérez-Pascual et al., 2017).

Diverse lytic functions were also identified at high abundance in the *T. maritimum* secretome. The tissue-degrading enzymes including chondroitinase ClsA (A0A5S9V218), sialidase SiaA (A0A5S9REV9), sphingomyelinase Sph (A0A5S9TEZ1), ceramidase Cer (A0A5S9U028), and collagenase Col (A0A5S9TI68) were exclusively found in the S-ECPs (**Supplementary Table S2**). Other predicted lytic functions including some proteases such as A0A5S9SSW8, ClpP (A0A5S9W994), and PtrB (A0A5S9U355) and a thioesterase (A0A5S9S238) were also identified in S-ECPs and not in OMVs. In addition, the pore-forming toxin tenacilysin Tly (A0A5S9THM7) was ca. 18-fold more abundant in the soluble fraction than in OMVs. The same happened with a lectin/glycoside hydrolase (A0A5S9RL88), a predicted adhesin (A0A5S9SKG2), or a type III fibronectin domain-containing protein (A0A5S9V4P1), which were up to threefold more abundant in S-ECPs than in OMV. By contrast, up to six probable degrading enzymes were found at the same levels in OMVs and S-ECPs. They include two metaloproteases (A0A5S9UZY9 and A0A5S9RFG6), peptidase AprN (A0A5S9SGZ5), serine protease DegP (A0A5S9VL96), and two putative lipase/esterases (A0A5S9US06 and A0A5S9RVG4). Some stress resistance functions were also identified in the ECPs. While the superoxide dismutases SodA (A0A5S9S479) and SodC (A0A5S9S8T8) were found in both ECP compartments at the same levels, catalase KatB (A0A5S9SXC2) was detected only in OMVs, and chaperon GroL (A0A5S9S9H1) was detected only in S-ECPs. All these results clearly show that most components of the *T. maritimum* secretome are specific to one or another ECP fraction. Thus, the differences found between the protein composition of S-ECPs and OMVs suggest that they should play specialized roles in *T. maritimum* biology or virulence.

### Analysis of proteolytic and lipolytic activities in the ECP fractions

**Figure 6:**
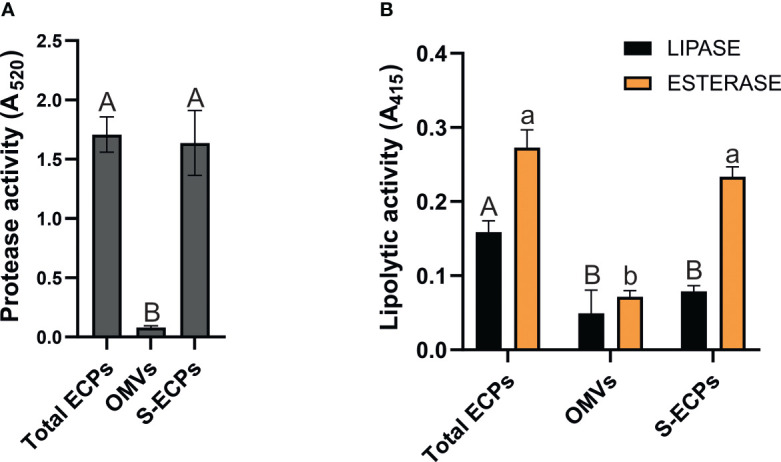
Proteolytic and lipolytic activities of *Tenacibaculum maritimum* ECP fractions. **(A)** Distribution of proteolytic activity in total extracellular products (ECPs), insoluble (outer membrane vesicles (OMVs)), and soluble extracellular products (S-ECPs) of *T. maritimum*. **(B)** Distribution of lipolytic activity (esterase and lipase) in ECPs, OMVs, and S-ECPs of *T. maritimum*. Bars represent the means ± SD (n = 3). Different letters denote significant variations between total ECPs, OMVs, and S-ECPs for each activity (single-factor ANOVA; *p*< 0.05).

Proteolytic and lipolytic activities of *T. maritimum* OMV and S-ECP fractions were quantified using commercial substrates and compared to those found in total ECPs. Azocoll was used to evaluate proteolytic activity. Hydrolysis of 4-nitrophenyl butyrate (*p*-NPB) was used to evaluate esterase activity and 4-nitrophenyl caprylate (*p*-NPC) for lipase activity. The results obtained are shown in **Figure 6**. OMV fraction showed residual proteolytic activity (0.081 ± 0.013). By contrast, total ECPs and S-ECPs showed effective azocoll hydrolysis at equal levels (1.71 *vs.* 1.65) (**Figure 6A**). This result greatly suggests that proteolytic activity is mainly associated with the soluble fraction of the *T. maritimum* ECPs.

When lipolytic activities were measured, quite different patterns of esterase and lipase activities were found. Lipase activity achieved 0.16 ± 0.02 in total ECPs and halved when evaluated in the OMV and S-ECP fractions. Statistical differences were not detected between OMV and S-ECP lipase activities. This result shows that the lipase activity of *T. maritimum* total ECPs is the sum of each fraction. Likewise, there were no statistical differences between the esterase activity of the ECPs and its soluble fraction (S-ECPs).

These results are congruent with the differential presence of several enzymes in each OMV and S-ECP proteome.

### Role of *T. maritimum* ECPs in biofilm promotion

**Figure 7:**
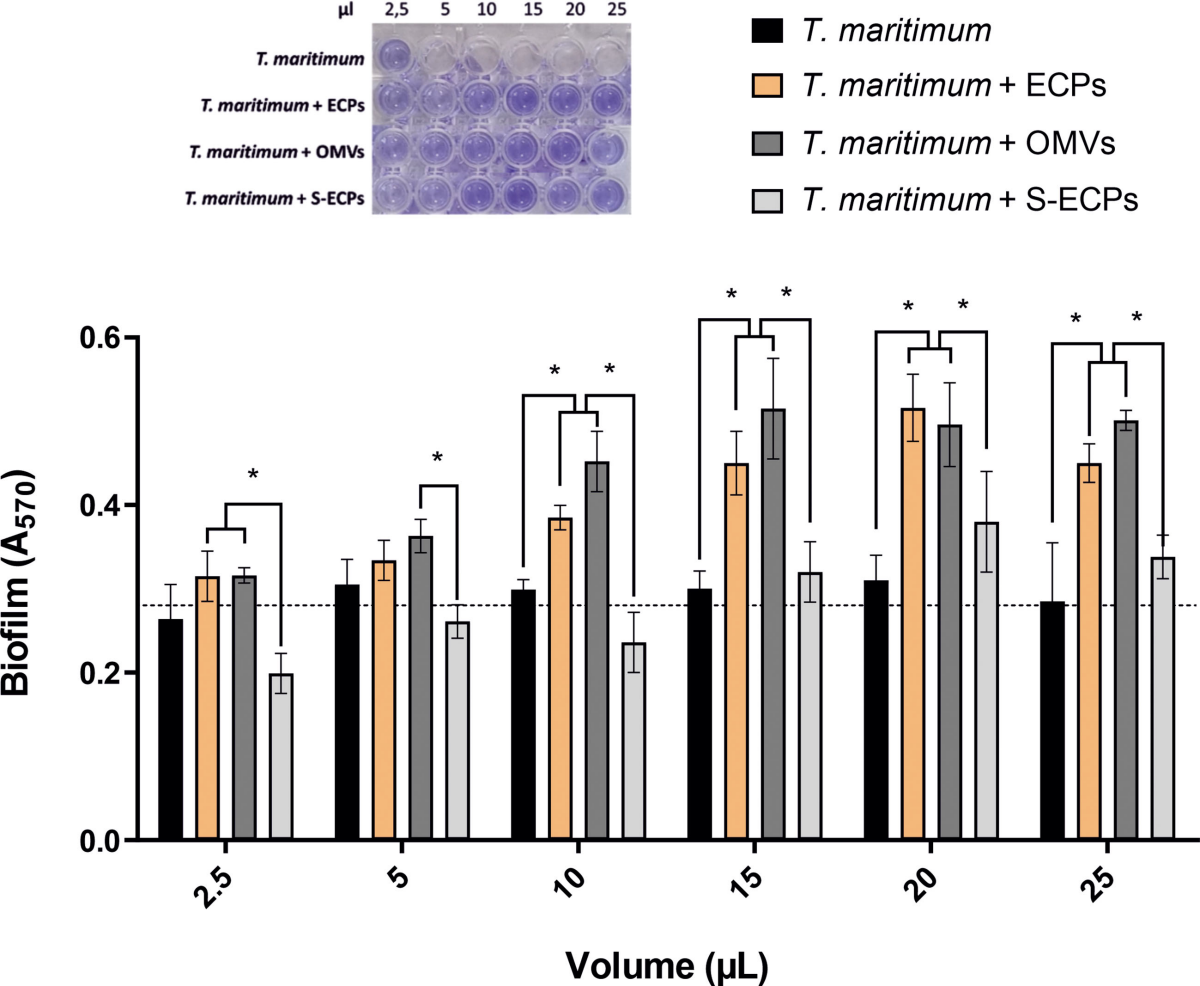
Formation of *in vitro* biofilm obtained from *Tenacibaculum maritimum* in presence of different extracellular products (total ECPs, OMVs, and S-ECPs). *T. maritimum* adhesion to polycarbonate surface and biofilm formation in normal conditions (black) and in presence of different volumes (2.5, 5, 10, 15, 20, and 25 µl) of ECP (orange), OMV (dark gray), and S-ECP (gray) extracts were quantified. Biofilm formation capacity was assayed under static conditions by crystal violet staining and subsequent determination of A_570_. Bars represent the means ± SD (n = 3). Asterisk denotes significant differences between experimental groups (two-way ANOVA; *p*< 0.05). Dotted line indicates the mean value of the controls. ECPs, extracellular products; OMVs, outer membrane vesicles; S-ECPs, soluble extracellular products.

*T. maritimum* SP9.1 strain produces a profuse biofilm (Levipan et al., 2019). To determine whether extracellular products play a role in *T. maritimum* active attachment and biofilm formation, biofilm production assay was performed using *T. maritimum* cultures supplemented with 2.5 to 25 µl at 0.5 mg/ml of total ECP, OMV, or S-ECP extracts. The controls of *T. maritimum* cultures supplemented with 2.5–25 µl of phosphate-buffered saline (PBS) reached an average biofilm production of 0.275 ± 0.014 (A_570_) after 5 h (**Figure 7**). The addition of total ECPs promoted biofilm formation, achieving maximal biofilm production of ca. A_570 _= 0.5 when supplemented with 20 µl of total ECPs. Interestingly, similar results were obtained when *T. maritimum* was supplemented with OMVs. No differences were found between the controls supplemented with ECPs and those with OMVs. In both cases, the addition of 10 µl or more significantly enhanced biofilm production in a dose-dependent manner. By contrast, the addition of just 2.5 µl of S-ECP extract appears to reduce the formation of biofilm. The results clearly suggest that production of OM vesicles helps *T. maritimum* to produce biofilm.

### *T. maritimum* ECPs produce toxic effects on fish cell lines and lesions on fish skin

**Figure 8:**
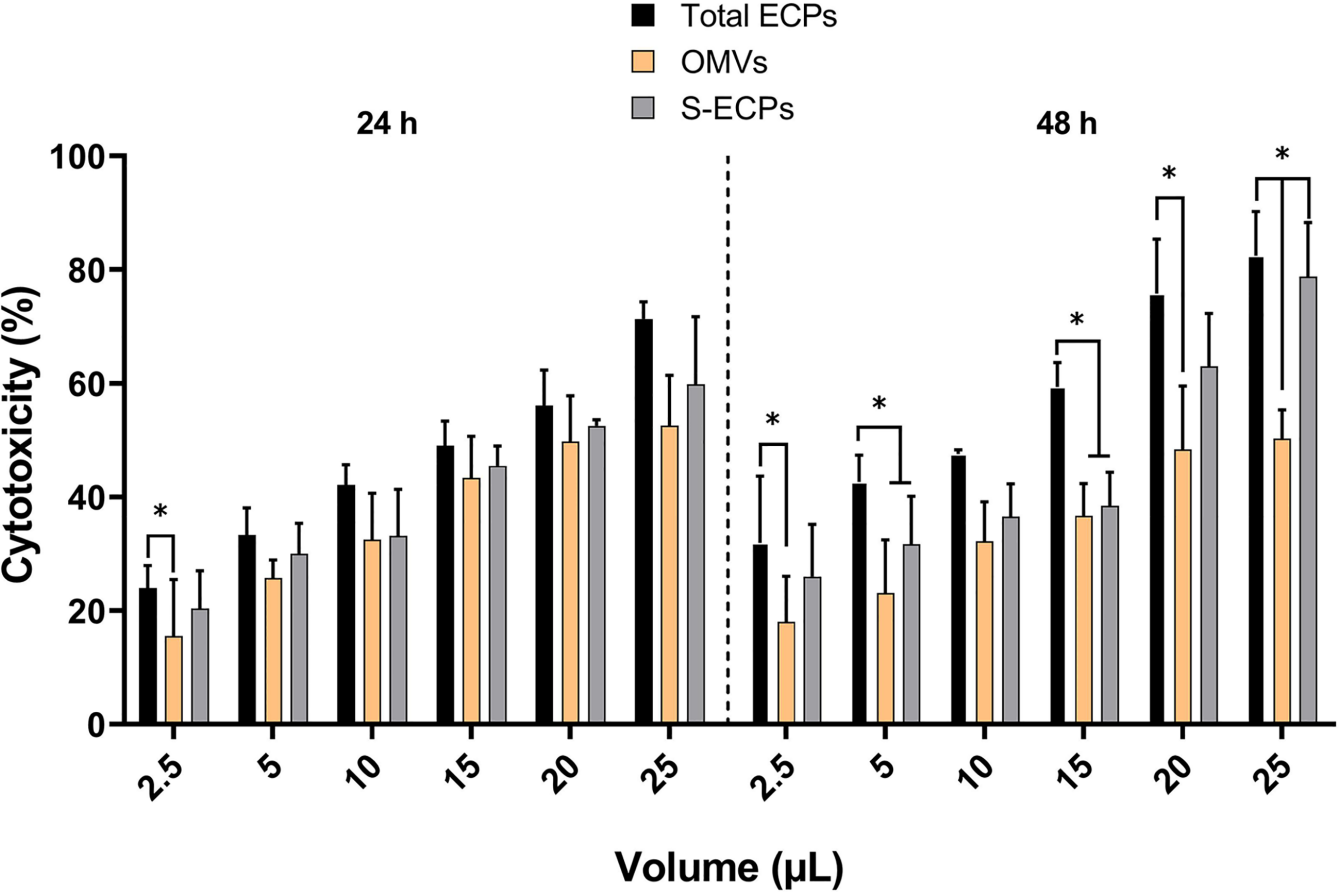
Percentage of cytotoxicity or non-viable cells with respect to the control group after 24 and 48 h of exposure of epithelioma papulosum cyprini (EPC) cells to *Tenacibaculum maritimum* total ECPs, OMVs, and S-ECPs, expressed as mean ± SD (n = 3). ECPs, extracellular products; OMVs, outer membrane vesicles; S-ECPs, soluble extracellular products. Asterisk (*) denotes significant differences between experimental groups (two-way ANOVA; p < 0.05).

Epithelioma papulosum cyprini (EPC) cell line and Senegalese sole fingerlings were used to study the toxicity of the extracts. All the extracts showed dose-dependent cytotoxic effects for the cell line at all concentrations tested. The cytotoxic effect that was measured as the percentage of viable cells correlated with the microscopic observation of alteration and destruction of the cell layer (**Supplementary Figure S3**). After 24 h of treatment, the percentage of non-viable cells at the lowest concentration was just 24% in the samples supplemented with total ECPs (**Figure 8**). The cytotoxicity increased in a concentration-dependent manner at 24 and 48 h (**Figure 8**). The cytotoxicity effect was higher after the addition of total ECPs than when OMV or S-ECP fractions were added. After 48 h of treatment, total ECPs showed significantly more cytotoxic effects than OMVs alone (**Figure 8**). In addition, cell cultures treated with 10 to 15 µl of total ECPs were also significantly more destroyed than those treated with OMV or S-ECPs (**Figure S3**). Interestingly, S-ECPs tended to induce higher cytotoxic effects, specifically after 48 h and high doses. However, statistical differences were only observed between total ECP and OMV treatment and after 48 h of exposure.

**Figure 9:**
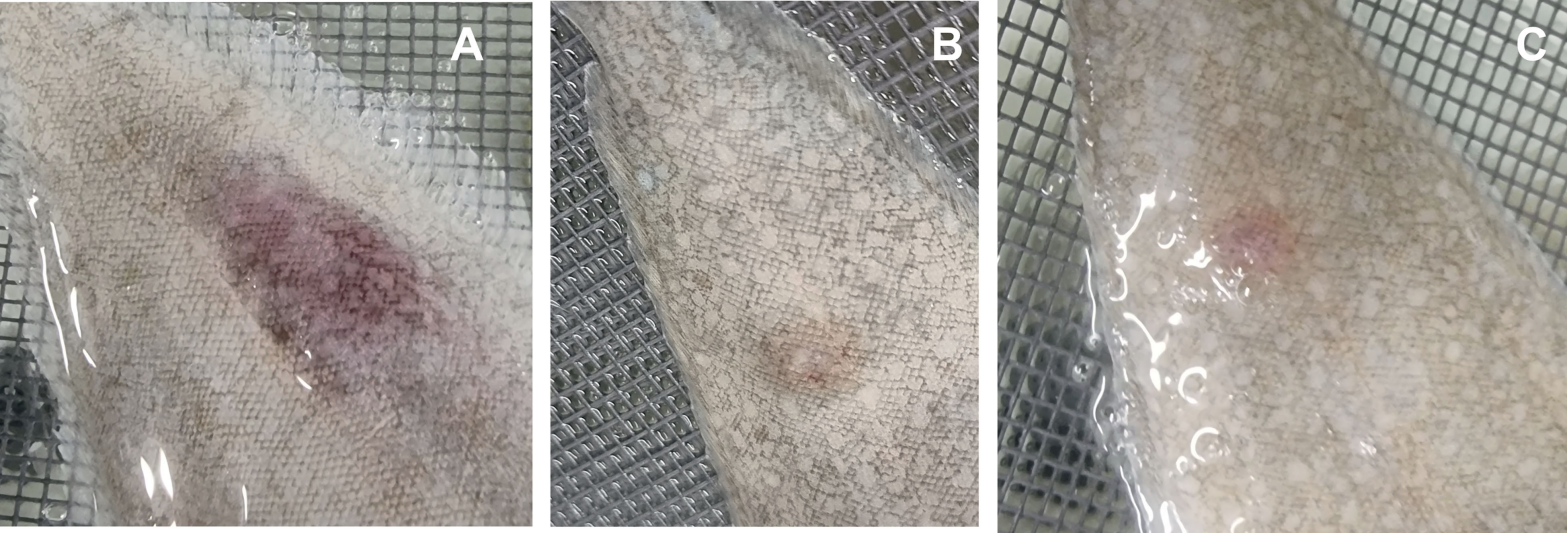
Ulcerative and hemorrhagic lesions produced by total ECPs **(A)**, OMVs **(B)**, and S-ECPs **(C)** of *Tenacibaculum maritimum* on the skin of Senegalese sole (*Solea senegalensis*). ECPs, extracellular products; OMVs, outer membrane vesicles; S-ECPs, soluble extracellular products.

Finally, sole (*Solea senegalensis*) fingerlings were subcutaneously injected with total ECPs or one of the fractions (either OMVs or S-ECPs). Representative images of the qualitative effects observed are shown in **Figure 9**. Total ECPs from *T. maritimum* showed higher toxic effects for sole, causing ulcerative and hemorrhagic lesions between 12 and 24 h after inoculation. Apparently, toxic effects were lower in the fish injected with S-ECPs (**Figure 9C**) or with OMVs (**Figure 9B**). These results suggest that S-ECPs and OMVs likely play different roles in virulence and that some unidentified ECP components are needed to achieve maximal toxic effects in fish.

## Discussion

ECPs are important virulence factors for many fish pathogenic bacteria including *T. maritimum* (Magariños et al., 1992; Newton et al., 1997; Zorrilla et al., 2003; van Gelderen et al., 2009; Labella et al., 2010). *T. maritimum* pathogenesis is mainly attributed to the synergistic interaction of ECP toxins and enzymes, which allows the pathogen to invade the host, causing extensive damage in the host tissues (Baxa et al., 1988; Magariños et al., 1995; Pazos, 1997; Pérez-Pascual et al., 2017). Although *T. maritimum* is considered a highly homogeneous species due to its phenotypical characteristics (Bernardet et al., 1994; Pazos, 1997), four O serotypes have been defined (Avendaño-Herrera et al., 2005). Most strains characterized to date belong to serotypes O1, O2, and O3. Serotype O4 strains have been isolated only occasionally from diseased fish (Avendaño-Herrera et al., 2005; Pazos, 1997) during the last decade, although recent epidemiological studies show an increasing incidence of outbreaks caused by O4 strains (Santos, 2022). It is likely that the use of vaccines against fish tenacibaculosis (mainly formulated with strains of the serotype O1/O2/O3) would be exerting a selective pressure, which would result in an increased incidence of tenacibaculosis outbreaks caused by serotype O4 (Santos, 2022). In the present work, a collection of 64 strains with diverse geographic origins, hosts, and serotypes was used to study the prevalence of some hydrolytic enzymatic activities related to virulence present in *T. maritimum* ECPs. All strains belonging to serotypes O1–O3 showed identical enzymatic patterns, which reinforces the idea that *T. maritimum* is highly homogeneous in this regard. However, 16 strains belonging to serotype O4 that were isolated from diseased fish mainly from 2013 to date showed unexpected heterogeneity in their profiles of ECP enzymatic activity. Thus, we selected the serotype O4 strain SP9.1 to characterize the protein content of its ECPs and use it as a model to analyze the *T. maritimum* secretome. The ECPs were obtained under conditions of low iron availability to ensure the expression of most virulence factors (Cassat and Skaar, 2013), and their protein content was analyzed through the high-throughput nLC-TIMS-QTOF proteomic strategy, which allowed the identification of 641 proteins. Two different fractions were found in these ECPs, a soluble fraction that contained several enzymatic activities and in which 264 non-redundant proteins were identified and an insoluble fraction containing 65 specific proteins. A total of 166 proteins were common in both fractions.

The monitoring of *T. maritimum* attachment to glass coverslips and the TEM visualization of the insoluble fraction of the ECPs suggested that this fraction was formed by some types of outer membrane vesicles that could participate in surface colonization. Production of classical OMVs and long bleb structures was also visualized by TEM/SEM in *F. columnare*. This phenomenon could be connected to secretion systems, gliding motility, and the surface adhesion of the bacterial cells (Laanto et al., 2014). OMVs are nanospherical proteoliposomes released from the outer membrane of many Gram-negative bacteria, which mainly contain lipopolysaccharides, outer membrane proteins, and other specific proteins within their lumen (Schwechheimer and Kuehn, 2015; Toyofuku et al., 2019; Furuyama and Sircili, 2021; Mathivanan et al., 2021). *T. maritimum* ECP proteome characterization showed that they are exceptionally abundant in outer membrane proteins, including components of the T9SS, a high number of TBDTs, and lipoproteins. All these observations suggest for the first time that *T. maritimum* produces OMVs as part of its ECPs. It is noteworthy that the production of OMVs is associated with the presence of a functional T9SS, a newly identified secretion system widely distributed in the *Cytophaga*–*Flavobacterium*–*Bacteroides* cluster, which could be the main mechanism of virulence and likely aids the colonization of fish tissues (McBride, 2019). T9SS enables gliding motility, enhances adhesion and biofilm formation, and could secrete an array of virulence factors to the cell surface or the extracellular medium (McBride, 2001; McBride and Zhu, 2013; Veith et al., 2015). T9SSs are common in members of the phylum *Bacteroidetes* and have been reported in fish pathogens such as *F. columnare* or *Flavobacterium psychrophilum*, where mutants deficient in gliding motility or in secreted proteins show reduced virulence (Li et al., 2017; Barbier et al., 2020; Thunes et al., 2022; Thunes et al., 2023).

It is noteworthy that the genome of *T. maritimum* SP9.1 harbors 23 genes that would encode putative TBDTs. Interestingly, all their predicted gene products were found as part of the ECP content analyzed in this work. OMVs of *Acinetobacter baumannii* were also found selectively enriched in TonB-dependent transporters, playing a key role in iron acquisition (Dhurve et al., 2022). The presence of so many putative TBDTs suggests that *T. maritimum* could use siderophores produced by other bacteria, a phenomenon known as siderophore piracy (Cordero et al., 2012; Kümmerli et al., 2014; Kramer et al., 2020), to grow in fish tissues. This reinforces the idea that *T. maritimum* is a species that takes advantage of co-infections to colonize fish hosts. In addition, homologs of heme uptake functions, which were described as virulence factors since they are involved in heme uptake (Olczak et al., 2008), were also found in OMVs of *T. maritimum*. In *Bacteroidetes*, three distinct subtypes of TBDTs are presumably regulated in different ways to tune nutrient uptake other than iron (Pollet et al., 2021). In this sense, three TBDT homologs to SusC/SusD, which mediate starch utilization from the host in *Bacteroidetes* (Reeves et al., 1997), were found to be overrepresented in *T. maritimum* OMVs. However, in accordance with the inability of *T. maritimum* to use carbohydrates, these proteins were predicted to be involved in glycan harvesting from host glycoproteins (Pérez-Pascual et al., 2017; Mabrok et al., 2023). Furthermore, it cannot be ruled out that the high prevalence of outer membrane proteins in *T. maritimum* OMVs could enhance bacterial fitness by serving as a decoy mechanism against phage infections (Biller et al., 2014; Reyes-Robles et al., 2018). All these hypotheses must be further studied.

Furthermore, among the most abundant proteins of the ECPs, we found diverse hydrolytic enzymes including chondroitinase ClsA, sialidase SiaA, sphingomyelinase Sph, ceramidase Cer, and collagenase Col, which were proposed as virulence factors in *T. maritimum* (Pérez-Pascual et al., 2017). In congruence with the observed proteome of the S-ECPs, these enzymatic activities with the capacity to degrade tissues were almost exclusively found in the ECP soluble fraction. This supports the hypothesis that in *T. maritimum*, the secretion of toxins is one of the main virulence factors. This strategy could differ from other species of the genus, such as *Tenacibaculum dicentrarchi*, *Tenacibaculum ovolyticum*, or *Tenacibaculum soleae* (Grothusen et al., 2016; Lujan et al., 2016; Teramoto et al., 2016) since most of the predicted toxins present in *T. maritimum* (tenacilysin, collagenase, sphingomyelinase, ceramidase, sialidase, and chondroitin AC lyase) are absent from the genomes of the other species. However, our results also evidenced that both compartments (S-ECPs and OMVs) are needed together to achieve maximum lytic levels. Thus, the extracellular products of *T. maritimum* must be enriched with OMVs also loaded with hydrolytic enzymes. The differences in protein content found between S-ECPs and OMVs suggest that each fraction would play specialized roles in *T. maritimum* biology and virulence. OMVs would play a relevant role in the formation of biofilm and esterase and lipase lipolytic activities. Both fractions were cytotoxic for fish cell lines and caused ulcerative and hemorrhagic lesions in Senegalese sole. However, cytotoxicity effects were higher in S-ECPs than in OMVs, being the sum of both what causes a higher degree of fish cell alteration.

It is well reported that *T. maritimum* generates a profuse biofilm (Mabrok et al., 2023). Our results greatly suggest that the OMVs produced by this bacterium play a major role in adhesion and biofilm formation. In addition, the characterization of OMV-associated proteins also showed that OMVs would serve as a secretion vehicle since the addition of total ECPs and OMVs to the *T. maritimum* culture showed a significant increase in biofilm formation. For that reason, these extracellular structures are considered by some authors as a new secretion system “type zero” (Kieselbach et al., 2015; Guerrero-Mandujano et al., 2017; Macion et al., 2021). Thus, the production of OMVs would help *T. maritimum* to increase its capacity to form biofilms. Initial adhesion to host cells is a key factor for bacterial colonization and pathogenicity, and it has been previously reported in several pathogens of the phylum *Bacteroidetes*, such as *Flavobacterium johnsoniae*, *F. psychrophilum*, or *F. columnare* and in other pathogens such as *Aeromonas hydrophila* (McBride and Nakane, 2015; Li et al., 2017; Barbier et al., 2020; Seike et al., 2021). In addition, the capability to produce a profuse biofilm allows *T. maritimum* to persist in the aquatic environment where surfaces of aquaculture facilities and fish mucus can serve as reservoirs for this bacterium (Levipan et al., 2019). Our results are in line with those results on other *Bacteroidetes* species. Interestingly, some works found that mucus from the skin of fish infected with *T. maritimum* shows a delay in the immune response compared to that found at the systemic level (Guardiola et al., 2019; Escribano et al., 2020). OMVs of *F. psychrophilum* contain important antigenic proteins and may play a possible role in the interaction with the host immune system (Møller et al., 2005). OMVs of *F. columnare* were found to contain diverse proteins with unknown functions and an OmpA-family protein, which is associated with virulence in other bacterial pathogens (Arias et al., 2012; Laanto et al., 2014). ECPs and OMVs are also being developed as putative vaccines against different bacterial species (Ellis and Kuehn, 2010; van Gelderen et al., 2010; Park et al., 2011; van de Waterbeemd et al., 2013; Acevedo et al., 2014; Teixeira et al., 2023).

To the best of our knowledge, this is the first description of the production of outer membrane vesicles by *T. maritimum* and their role in virulence. Our findings clearly show that virulence-associated properties such as active surface attachment, biofilm formation, and the production of lytic enzymes are closely related to the production of OMVs. However, most components of the *T. maritimum* secretome are specific to one or another ECP compartment. All extracellular products, OMVs specifically, could play a relevant role in future works aimed at the development of novel treatments and novel vaccines against tenacibaculosis in fish.

## Materials and methods

### Bacterial strains

All bacterial strains used in this study are listed in **Table S1** (**Supplementary Material**). *T. maritimum* strain SP9.1 was isolated from diseased Atlantic salmon in Spain and selected as the experimental model. Stock cultures were stored frozen in *Flexibacter maritimus* medium (FMM; Condalab) with 8% glycerol at −80°C in single-use cryovials (Ward and Watt, 1971).

### Enzymatic activity evaluation

The ability of *T. maritimum* to degrade some substrates was evaluated *in vitro* by radial diffusion method on FMM agar containing sterilized gelatine (0.6%), casein (0.5%), or chondroitin sulfate A (20%) (Sigma-Aldrich, Gillingham, UK) as previously described (Madsen and Dalsgaard, 1998; Li et al., 2015). Briefly, live whole cells of 64 strains of different origins, hosts, and serotypes (**Supplementary Table S1**) were plated in duplicate into FMM plates and incubated at 25°C for a minimum of 4 days before noting the appearance of clear zones. To enhance positive results, plates of gelatine were flooded with 15% (w/v) mercuric chloride in 20% (v/v) HCl. Degradation of chondroitin was visualized by flooding the plates with 1 M of HCl followed by incubation for 5 min. *T. soleae* CECT 7292 was used as a negative control. In addition, the ability of *T. maritimum* to degrade lipids was analyzed on FMM plates containing 1% Tween 20 (for esterase activity) or 1% Tween 80 (for lipase activity). The production of phospholipase was detected on plates of FMM containing egg yolk emulsion (Oxoid) as previously described (Pazos, 1997). After incubation of whole live cells of the same 64 strains for at least 48 h, the appearance of an opaque halo (calcium oleate crystals) around the extracts indicated a positive result. Hemolytic activity was evaluated by the standard radial diffusion method using an FMM medium with 5% (v/v) sheep erythrocytes added. A positive result was defined when the bacteria produced a zone of clearing equally in the area to that produced by 0.1% sodium dodecyl sulfate (SDS) (Bandín et al., 1991). Drops (10 μl) of each sample (total ECPs, OMVs, and soluble ECPs; see “ECP isolation and fractionation” below) were also deposited on plate surfaces in all conditions.

### Electron microscopy observation

The presence of OMVs in cells of *T. maritimum* strain SP9.1 was examined by SEM. SEM sample preparation was performed according to a previously described method with some modifications (Choi et al., 2015). *T. maritimum* was grown until the exponential phase (48 h at 25°C) in flasks containing 10 ml of FMM. Some glass coverslips were included within the medium to be colonized by the bacterium. A coverslip was removed from the flask at 10 and 24 h of incubation, and the bacterial cells attached to its surface were fixed in 2% (v/v) glutaraldehyde in 0.1 M of cacodylate buffer (pH 7.4). Then, samples were post‐fixed with 1% osmium tetroxide for 2 h. Finally, they were dehydrated with a series of graded ethanol solutions, starting with 50% and followed by 70%, 75%, 96%, and 100% (×3), and they were air-dried after a final dehydration overnight. Samples were sputter‐coated with iron and then examined by SEM (FESEM Ultra plus, ZEISS, Jena, Germany) at an acceleration voltage of 3 kV and a magnification of ×50,000. Subsequently, OMV extracts (see “ECP isolation and fractionation” below) were also observed by TEM. OMVs were placed on carbon-coated Formvar grids (EMS, USA) and negatively stained for 60 s with 2% uranyl acetate. The grids were observed by TEM (JEM1010, Japan) at an acceleration voltage of 80 kV and a magnification of ×50,000.

### ECP isolation and fractionation

Total ECPs, OMVs, and soluble ECPs were isolated following the cellophane overlay method as described previously with some modifications (Liu, 1957; Kadurugamuwa and Beveridge, 1995; Klimentová and Stulík, 2015). Briefly, cultures of 50 ml of *T. maritimum* strain SP9.1 were grown at 25°C for 24 h in FMM, supplemented with the iron chelator EDDA (Sigma-Aldrich) at 20 µM. Two plates of FMM agar (23 × 23 cm) with the sterile cellophane overlay (Pacon) were incubated with the cell cultures for 48 h at 25°C, one plate for total ECPs and another one to separate it in two fractions: OMVs and soluble ECPs. This also allowed to minimize growth-phase effects since the different proteases are produced at different times of the growth curve (Gudmundsdóttir, 1996). Bacteria of each plate were washed off the cellophane using 40 ml of PBS (pH 7.4) and centrifuged at 4,000 rpm for 30 min, and the pellet was discarded. Supernatants of the first plate were sequentially filtered through 0.45‐ and 0.2‐μm membrane filters (Millipore, Billerica, MA, USA), resuspended in 10% trichloroacetic acid (TCA), incubated for 2–3 h in ice to precipitate total ECPs, and stored in small volumes at −20°C until use. Cell-free supernatants of the second plate were centrifuged to collect the vesicles as a pellet by ultracentrifugation using a Beckmann SW32Ti rotor at 24,000 rpm at 4°C for 2 h. OMVs (the pellet) were resuspended in urea 7 M, 20 mM of Tris–HCl pH 8.0. The supernatant (soluble fraction of the ECPs and S-ECPs) was sterilized using 0.22-μm filters (Millipore), resuspended in 10% TCA, and incubated for 2–3 h in ice to precipitate soluble ECPs. S-ECPs were centrifuged at 15,000 rpm for 30 min, resuspended in urea 7 M, and stored at −20°C for further experimentation (**Supplementary Figure S1A**). Total ECPs and soluble ECPs used to test enzymatic activities were directly filtered and stored without precipitation with TCA to prevent protein denaturation and loss of functional activity (**Supplementary Figure S1B**). Bacterial cultures and purification procedures were made in triplicate to evaluate the reproducibility of the results. Total ECPs, OMVs, and soluble ECPs were plated in FMM plates to verify that samples were cell‐free. The protein content of total ECPs, OMVs, and soluble ECPs was determined with a Bradford assay kit (Bradford, 1976) using bovine serum albumin as a standard (Bauman and Kuehn, 2006) to reach a final concentration of 0.5 mg/ml in all samples.

### LC-MS/MS analysis and label-free quantification

The protein content of three individual biological replicates of ECPs, OMVs, and S-ECPs was analyzed by LC-MS/MS. Trypsin digestion and LC-MS/MS analysis were performed by the Mass Spectrometry and Proteomics Service of the University of Santiago de Compostela, as follows. Each protein sample (4 μl) was trypsin-digested using *In-Solution Tryptic Digestion and Guanidination Kit* (Thermo Fisher Scientific, Waltham, MA, USA). Reduction, alkylation, and digestion procedures were performed according to kit instructions, omitting the guanidination step. The resulting peptide mixtures were desalted by ZipTip C18 (Millipore) and diluted at 50 ng/μl in 0.1% formic acid. Peptide samples were analyzed on an ultrahigh-pressure nanoflow chromatography system (nanoElute, Bruker Daltonics, Billerica, MA, USA) coupled to a trapped ion mobility quadrupole time-of-flight mass spectrometer (timsTOF Pro, Bruker Daltonics) via a nanoelectrospray ion source (Captive Spray Source, Bruker Daltonics). Peptides were loaded and separated on an analytical column ReproSil C18 column (150 × 0.075 mm, 1,9 µm, 120Å) (Bruker FIFTEEN) with a sample injection volume of 2 μl. Mobile phases were water and acetonitrile buffered with 0.1% formic acid. MS spectra were collected under the following parameters: tims ramp time 100 ms, PASEF on, 200 ms, scan range (*m*/*z*, 100−1,700; 1/k0, 0.65−1.45 V·s/cm^2^).

The accession, visualization, and further analysis of LC-TIMS-Q-TOF data were provided by the manufacturer’s closed-source library, integrated into Bruker’s proprietary software DataAnalysis (Compass HyStar 5.1, otofControl 6.2 and DataAnalysis 5.3). Proteomics data were curated to remove common contaminants (e.g., keratins and trypsin) using the Contaminants MaxQuant1.6.17.0 database. Data acquired (mass/charge ratios) were compared against peptide MS/MS spectra of the *T. maritimum* SP9.1 theoretical proteome. The spectra were searched with the following parameters: peptide mass tolerance 15 ppm, fragment tolerance 0.02 Da, enzyme set as trypsin and allowance up to two missed cleavages, fixed modification of carbamidomethylation (+57 Da), and dynamic modification of acetylation (Protein N‐term, +42.01 Da), deamidation (+0.98 Da), oxidation (+15.99 Da), or dehydration (−18.01 Da). A protein was unequivocally identified if a minimum of two unique peptides matched it. For each protein identified, the predicted subcellular location, and Clusters of Orthologous Groups of proteins (COG) and KEGG groupings were established using eggNOG-mapper tool version 5.0 (http://eggnog-mapper.embl.de/). Venn diagram was used to visualize numeric data of presence/absence and overlaps between proteins lists identified in each subcellular preparation (ECPs, OMVs, and S-ECPs).

PEAKS Studio 10.6 (Bioinformatics Solutions, Waterloo, ON, Canada) was used to study relative peptide abundances. PEAKS Q Module for label-free quantification was used to calculate peptide peak areas (sum of peak areas for each charge state). The Q module mass error tolerance was set to 20.0 ppm, the false discovery rate (FDR) threshold was set to 1%, the retention time shift tolerance was set to 0.05 1/k0, and the alignment of the retention time was set. Peptides and peak areas were TIC (total ion count) normalized. One-way ANOVA was used to identify proteins differentially present in OMVs or S-ECPs. A double criteria of *p*-values<0.05 and fold change cutoff of ≥2 were used to assume the existence of statistical differences. Quantification values of differentially expressed proteins were visualized as a heat map using GraphPad Prism 9.0 software. Functional annotations and keywords of identified proteins were adopted from UniProt Knowledgebase (http://www.uniprot.org).

The mass spectrometry proteomics data have been deposited to the ProteomeXchange Consortium via the PRIDE (Pérez-Riverol et al., 2022) partner repository with the dataset identifier PXD041510.

### Proteolytic activity quantification

For the quantification of the total proteolytic activity present in the total ECP, OMV, and soluble ECP samples, a general proteolytic substrate (Azocoll, Sigma) was employed as previously described (Magariños et al., 1992). Briefly, 0.02 g of azocoll was dissolved in 2.5 ml of Tris-Cl 0.1 M pH 7.2, and 100 μl of the sample was added and incubated at 37°C for 1 h. The reactions were stopped by adding 2.5 ml of TCA 10%, centrifuged 2,000 ×*g* for 15 min at 4°C, and measured using a spectrophotometer (Hitachi U200) at 520 nm. PBS was used as blank. One unit of protease activity (1 U) was defined as the increase of 0.1 in the absorbance value at 520 nm due to azocasein hydrolysis.

### Lipolytic activity quantification

Esterase and lipase activities were quantified by following the hydrolysis of *p*-nitrophenyl esters and measured spectrophotometrically at 415 nm (De Yan et al., 2013). Substrate *p*-NPB for esterase activity and substrate 4-*p*-NPC for lipase activity were prepared as follows. *p*-NPB solution was prepared by adding 0.1 ml of solution A (10 mM of *p*-NPV in acetonitrile) into 2.8 ml of solution B (50 mM of Tris–HCl buffer, pH 7.4). *p*-NPC solution was prepared by adding 0.1 ml of solution A (10 mM of *p*-NPV in acetonitrile) into 2.8 ml of solution C (2.22 g of Triton X-100 in 500 ml of 50 mM of Tris–HCl buffer, pH 7.4). The *p*-NPB and *p*-NPC solutions were incubated at 37°C for 15 min. For each enzymatic activity assay, 10 µl of the sample and 190 μl of the reaction mixture were mixed in 96-well plates and incubated at 37°C for 10 min. Finally and immediately, the absorbance was measured at 415 nm. Blank reactions were used for each measurement to subtract the non-enzymatic hydrolysis. All assays were performed in triplicate and represented as the mean value resulting from subtracting the kinetics of a given denatured sample under the same conditions and including the standard deviation. One enzyme unit (U) was defined as the increase in 0.1 absorbance at 415 nm due to *p*-nitrophenol release.

### Active attachment and biofilm formation assay

Active attachment and biofilm formation were quantified in flat-bottom, 96-well polystyrene plates (Corning Costar, New York, NY, USA), using a modification of a standard crystal violet staining procedure (O’Toole and Kolter, 1998). Briefly, *T. maritimum* SP9.1 cells from an FMM plate incubated for 48 h were harvested in 7 ml of FMM broth at 25°C for 24 h; 200 μl of the culture was transferred to a 96-well plate containing 25, 20, 15, 10, 5, and 2.5 μl of each extract (total ECPs, OMVs, and S-ECPs) at a protein concentration of 0.5 mg/ml and incubated at 25°C for 5 h. Negative control wells contained the *T. maritimum* culture without any ECP extract. The plate was then scanned in an iMark microplate reader (Bio-Rad, Hercules, CA, USA) to determine the OD_600_. Immediately, the cultures were aspirated out of the plate, and each well was fixed with 200 μl of methanol. The wells were then stained with 200 μl of 0.1% crystal violet and incubated at room temperature for 10 min. After incubation, the crystal violet was removed, and the wells were washed six more times with 150 μl of dH_2_O. The crystal violet remaining was solubilized with 200 μl of 30% acetic acid, and the A_570_ was measured in a spectrophotometer. The experiment was carried out in triplicate. Results are reported as relative A_570_ values (averages of three replicates; normalized to the highest average in a dataset or the wild type).

### Cytotoxicity assays

The ECP preparations (total ECPs, OMVs, and soluble ECPs) were assayed for cytotoxicity using the fish cell line EPC and the MTT assay, which allows to determine the viability of eukaryotic cells from mitochondrial activity. The assay is based on the conversion of the water-soluble yellow dye MTT (3-[4,5-dimethylthiazol-2-yl]-2,5-diphenyltetrazolium bromide) into formazan crystals by the action of mitochondrial reductases (van Meerloo et al., 2011). Monolayers were grown in minimum essential medium Eagle with Earle’s salts (EMEM; Sigma-Aldrich, UK) supplemented with 10% fetal bovine serum (Lonza, Pontevedra, Spain) and antibiotics (100 IU/ml of penicillin and 100 μg/ml of streptomycin (EMEM-10)) and then incubated at 25°C in 96-well plates for 24 h. When a semi-confluent monolayer (~80%) was reached, cells were inoculated with 25, 20, 15, 10, 5, and 2.5 μl of each ECP extract (three wells per concentration) at the same protein concentration (0.5 mg/ml) and incubated at 15°C for 24 or 48 h. Ten microliters of PBS and 10 μl of dimethyl sulfoxide (DMSO) (Sigma-Aldrich) were used as negative (0% lysed cells) and positive (100% lysed cells) controls, respectively. After microscopic examination, the MTT assay was performed. First, the medium was removed and replaced with 100 μl of PBS. To each well, 10 μl of 12 mM MTT (Acros Organics, Thermo Fisher Scientific) stock solution (5 mg/ml) was added. Plates were incubated at 25°C for 4 h. Then, the medium was removed, and formed formazan was solubilized by adding 100 μl of DMSO, and the concentration was determined by A_570_. All assays were performed in technical and biological triplicates. Results were expressed as mean ± SD. The percentage of non-viable cells or cytotoxicity was calculated as follows:


$$Cytotoxicity\;\left(\%\right)=100-\left(\;\frac{A_{570}-A_{positive\;control}}{A_{negative\;control}}*100\right).$$

### *In vivo* toxicity

The lethal effects of total ECPs, OMVs, and soluble ECPs produced by *T. maritimum* were evaluated in Senegalese sole (*S. senegalensis*) juveniles by i.p. inoculation of 0.1 ml of each preparation (van Gelderen et al., 2009) at the same concentration used for the virulence assays of live cells. Three groups of six fish were injected with each extract and maintained in tanks containing seawater (salinity, 32 PPT; water temperature, 26°C–27°C). Lesions were monitored daily over 7 days. Control fish were injected with 0.1 ml of PBS. All protocols for animal experimentation used in this study have been reviewed and approved by the Bioethics Committee of the University of Santiago de Compostela (approved protocol No. 15004/2022/003).

## Data availability statement

The data presented in this study are deposited in the PRIDE repository, accession number PXD041510.

## Ethics statement

The animal study was reviewed and approved by Bioethics Committee of the University of Santiago de Compostela (Protocol No. 15004/2022/003).

## Author contributions

All authors conceived and designed the study. ME performed the lab experiments. ME and MB analyzed the data and wrote the first draft of the manuscript. AT, BM, and ML obtained the funding resources. BM and ML corrected the draft and created the final version of the manuscript. All authors contributed to the article and approved the submitted version.

## Supplementary material

Supplementary Table S1 Tenacibaculum maritimum strains used in this study (n=64).

Supplementary Table S2 Proteins identified in both soluble (S-ECPs) and insoluble (OMVs) fraction of Tenacibaculum maritimum SP9.1 extracellular products (ECPs) related to virulence and/or host tissues destruction.

Supplementary Table S3 Presence/absence analysis and coverage area of the peptides that appeared in each sample compared with the SP9.1 genome.

Supplementary Table S4 Relative abundance of the proteins that appeared in both fractions analyzed to infer their location into each ECPs fraction.

Supplementary Figure S1 Preparation workflow. Total Extracellular products extraction (Total ECPs) and sub-cellular fractionation in outer membrane vesicles (OMVs) and Soluble extracellular products (S-ECPs) for proteomic analysis **(A)** and enzymatic activities assays **(B)**.

Supplementary Figure S2 Protease, lipolytic and haemolytic activities of Tenacibaculum maritimum SP9.1 cells and outer membrane vesicles in FMM plates supplemented with different substrates.

Supplementary Figure S3 Cytotoxicity effect of OMVs and S-ECPs from T. maritimum in EPC cell lines. Magnification, 30X.
