# Supplementary material for: The secretome of the fish pathogen Tenacibaculum maritimum includes soluble virulence-related proteins and outer membrane vesicles
Source: Front Cell Infect Microbiol. 2023 Jun 9;13:1197290. doi: 10.3389/fcimb.2023.1197290 (PMC10288586; doi:10.3389/fcimb.2023.1197290)
Supplement: Supplementary Table S1 — Tenacibaculum maritimum strains used in this study (n=64). [file DataSheet_1.pdf]

SUPPLEMENTARY

Table S1. *Tenacibaculum maritimum* strains used in this study (n=64)

STRAIN	SEROTYPE	HOST	ORIGEN	YEAR
NCIMB 2154^T	O1	*P. major*	Japón	1977
CA63.1	O1	*S. senegalensis*	España	2007
PC503.1	O1	*S. senegalensis*	España	2001
PC529.1	O1	*S. senegalensis*	España	2002
PC538.1	O1	*S. aurata*	España	2002
LM5.1	O1	*S. maximus*	España	2007
LM4.1	O1	*S. maximus*	España	2007
AF37.1	O1	*P. bogaraveo*	España	2006
AF39.1	O1	*P. bogaraveo*	España	2006
NCIMB 2153	O1	*A. schlegelii*	Japón	1976
COM8.1	O1	*S. senegalensis*	España	2006
IEO19.1	O1	*S. senegalensis*	España	2006
IEO26.1	O1	*S. senegalensis*	España	2006
RPM808.1	O1	*S. maximus*	España	2004
PC824.1	O1	*S. aurata*	España	2003
PC833.1	O1	*S. aurata*	España	2003
SNW20.1	O2	*S. salar*	España	2015
CA80.1	O2	*S. maximus*	España	2007
ACR485.1	O2	*S. senegalensis*	España	2011
ACR570.1	O2	*S. senegalensis*	España	2014
PC424.1	O2	*S. maximus*	España	2019
AC6.1/06	O2	*S. senegalensis*	España	2006
ACC102.1	O2	*S. maximus*	Portugal	2007
ACR567.1	O2	*S. senegalensis*	España	2014
RM285.1	O2	*S. maximus*	España	2006
PC682.1	O2	*S. maximus*	España	2003
PC1012.1	O2	*S. maximus*	España	2008
NCIMB 2158	O2	*S. solea*	Escocia	1981
TM12	O2	*D. labrax*	Francia	2010
SNW21.1	O2	*S. salar*	España	2015
SNW41.1	O2	*S. salar*	España	2017
CA43.1	O2	*S. senegalensis*	España	2006
SNW15.1	O3	*S. salar*	España	2014
CA56.1	O3	*S. senegalensis*	España	2007
AQP72.1	O3	*D. labrax*	España	2016
ACC13.1	O3	*S. senegalensis*	Portugal	2004
ACR568.1	O3	*S. senegalensis*	España	2014
COS3.1	O3	*S. senegalensis*	España	2011
SNW35.1	O3	*S. salar*	España	2016
AZ226.1	O3	*S. solea*	España	2010
AQP79.1	O3	*D. labrax*	Portugal	2016
SNW11.1	O3	*S. salar*	España	2014
ACC20.1	O3	*S. senegalensis*	Portugal	2004
CA4.1	O3	*S. maximus*	España	2015
ACC54.1	O3	*S. maximus*	Portugal	2006
RI136.1	O3	*S. maximus*	España	2002
ACC8.1	O3	*S. senegalensis*	Portugal	2003

STRAIN	SEROTYPE	HOST	ORIGEN	YEAR
SNW5.1	O3	*S. salar*	España	2014
VIL3.1	O4	*S. maximus*	España	2004
SNW24.1	O4	*S. salar*	España	2015
SNW17.1	O4	*S. salar*	España	2015
ACR685.1	O4	*S. senegalensis*	España	2018
ACR687.1	O4	*S. senegalensis*	España	2018
LSP69.1	O4	*S. senegalensis*	Portugal	2018
LSP110.1	O4	*S. senegalensis*	Portugal	2018
LSP9.1.1	O4	*S. senegalensis*	Portugal	2012
SP9.1.1	O4	*S. salar*	España	1993
SNW25.1	O4	*S. salar*	España	2015
AZ200.1	O4	*S. maximus*	España	2003
SNW20.2	O4	*S. salar*	España	2015
SE30.1	O4	*O. kisutch*	España	1993
LSP22.1	O4	*S. senegalensis*	Portugal	2013
LSP73.1	O4	*S. senegalensis*	Portugal	2013
LSP59.1	O4	*S. senegalensis*	Portugal	2013

Table S2. Proteins identified in both soluble (S-ECPs) and insoluble (OMVs) fraction of *Tenacibaculum maritimum* SP9.1 extracellular products (ECPs) related to virulence and/or host tissues destruction.

GENE NAME	PREDICTED FUNCTION	OMVs	SECPs	ACCESSION	LOC	COG	PFAM	SOURCE
**T9SS COMPONENTS/ GLIDING MOTILITY**								
GldJ	Gliding motility lipoprotein	+ (1.12)	+ (0.39)	A0A5S9SAF8_9FLAO	OM	N	FGE-sulfatase	Braun & McBride, 2005
PorK/GldK	OM lipoprotein, forms large periplasmic rings in association with PorN, PorM and PorL. Component of periplasmic channel.	+ (1.13)	+ (0.27)	A0A5S9SHI5_9FLAO	OM/ P	N	FGE-sulfatase	Braun et al., 2005
PorL/GldL	IM protein with two TMHs. Interacts with PorM, PorN and PorX	+ (N.Q)	-	A0A5S9TDY7_9FLAO	IM	N	-	Braun et al., 2005
PorM/GldM/GldO	IM protein with periplasmic domains. Component of periplasmic channel. Interacts with PorL, PorK and PorN.	+ (p>0.05)	+ (p>0.05)	A0A5S9SHI9_9FLAO	P/IM	N	GldM_C,GldM_ N	Braun et al., 2005
PorN/GldN	Forms large periplasmic rings in association with PorK.	+ (1.25)	+ (0.38)	A0A5S9SMJ7_9FLAO	OM/ P	N	-	Sato et al., 2010
PorP/SprF/SprP	Predicted 14-stranded OM β-barrel. Binds to PorE.	+	-	A0A5S9S662_9FLAO	OM	S	PorP_SprF,SPOR	Rhodes et al., 2011
PorT/SprT	Membrane protein. Predicted 8-stranded OM β-barrel protein	+	-	A0A5S9UQF7_9FLAO	OM	S	OMP_b-brl_2	Chen et al., 2011; Sato et al., 2005
PorU	Cell surface. Attachment complex, T9SS sortase, binds to PorV. Cysteine-type peptidase activity	+ (p>0.05)	+ (p>0.05)	A0A5S9RRK6_9FLAO	OM/ Cell	N	Peptidase_C25	Glew et al., 2012, 2017

					surface			
PorV	T9SS shuttle secreted proteins and attachment complex member. Binds to PorU, Sov, PorA, and other cargo	+ (0.43)	+ (0.62)	A0A5S9RRZ0_9FLAO	OM	IN	-	Chen et al., 2011 Kharade & McBride, 2015
**GLIDING MOTILITY/ ADHESION**								
SprA/Sov	Large OM protein	+	-	A0A5S9S815_9FLAO	OM	N	SprA_N	Nelson et al., 2007
SprB	Cell surface adhesin that is required for movement on agar	+ (0.44)	+ (1.35)	A0A5S9S4A4_9FLAO		MN	CHU_C,DUF11,HYR,IgGFc_binding,SprB	Nelson et al., 2008
SprC	Putative adhesin. C-terminal domain of CHU protein family	+	-	A0A5S9RKG3_9FLAO		N	CHU_C	
SprD	Cell surface protein	+ (p>0.05)	+ (p>0.05)	A0A5S9RHS8_9FLAO CAA0144965.1		TN	PorP_SprF,SPOR	
SprE	T9SS lipoprotein	-	-	A0A5S9UNF7_9FLAO		N	TPR_16,TPR_6,TPR_8	
SprF	T9SS protein	+ (1.75)	+ (0.22)	A0A5S9W5D1_9FLAO	OM	TM	PorP_SprF,SPOR	McBride and Zhu, 2013
Putative adhesin	Putative auto-transporter adhesin, head GIN domain	+ (0.16)	+ (0.96)	A0A5S9SKG2_9FLAO		N	DUF2807	
Putative adhesin	Fibronectin type-III domain-containing protein. Metallopeptidase activity.	+ (0.34)	+ (1.06)	A0A5S9V4P1_9FLAO		N	CHU_C,F5_F8_type_C,Glyco_hy	

	Por_Secre_tail domain-containing protein					dro_cc,PKD,PPC,Peptidase_M9, Peptidase_M9_N	
**ENZYMES**							
Lectin/glycoside hydrolase	Belongs to the glycosyl hydrolase 18 family containing a C-terminal secretion signal. Proteins displaying lectin or carbohydrate-binding motifs	+ (0.15)	+ (1.69)	A0A5S9RL88_9FLAO	G	PKD	Pazos, 1997
Protease, ClpP	ATP-dependent. Plays a major role in the degradation of misfolded proteins	-	+	A0A5S9W994_9FLAO	OU	CLP_protease	
Predicted protease	Probable S8/C10 family peptidase. Highly like streptopain containing a C-terminal secretion signal	-	+	A0A5S9SSW8_9FLAO	O	PA,PPC,Peptidase_M36	Nelson et al., 2011
Protease II (Oligopeptidase B), ptrB	oligopeptidase that cleaves peptide bonds following arginine and lysine residues	-	+	A0A5S9U355_9FLAO	E	Peptidase_S9,Peptidase_S9_N	
Metalloprotease	DUF11 domain-containing protein. Metallopeptidase activity.	+ (p>0.05)	+ (p>0.05)	A0A5S9UZY9_9FLAO	N	CHU_C,DUF11, WD40	
Metalloprotease	Probable M12B family metalloprotease containing a C-terminal secretion	+ (p>0.05)	+ (p>0.05)	A0A5S9RFG6_9FLAO	O	Reprolysin_4	
Peptidase AprN	Probable S8 family protease containing a C-terminal secretion signal	+ (p>0.05)	+ (p>0.05)	A0A5S9SGZ5_9FLAO	O	Peptidase_S8	Baxa et al., 1988
Serine protease DegP/htrA	Trypsin-like serine proteases, typically periplasmic, contain C-terminal PDZ domain	+ (p>0.05)	+ (p>0.05)	A0A5S9VL96_9FLAO	O	PDZ_1,PDZ_2,Trypsin_2	
Peptidase	M48 family metalloprotease	+	-	A0A5S9S0E6_9FLAO	O	Peptidase_M48	
Putative lipase	Alpha/beta hydrolase domain-containing protein putative lipase	+ (p>0.05)	+ (p>0.05)	A0A5S9US06_9FLAO	I	Abhydrolase_3	

Putative thioesterase	Thioesterase-like superfamily	-	+	A0A5S9S238_9FLAO	S	4HBT_2	
Esterase	Carbohydrate esterase family CE14	+ (p>0.05)	+ (p>0.05)	A0A5S9RVG4_9FLAO	G	PIG-L	
**STRESS RESISTANCE**							
60 kDa Chaperonin, groL	Prevents misfolding and promotes the refolding and proper assembly of unfolded polypeptides generated under stress conditions	-	+	A0A5S9S9H1_9FLAO	O	Cpn60_TCP1	
Superoxide dismutase [Mn/Fe], SodA	Destroys radicals which are normally produced within the cells, and which are toxic to biological systems	+ (p>0.05)	+ (p>0.05)	A0A5S9S479_9FLAO	P	CarboxypepD_reg,TonB_dep_Rec	Suzuki et al., 2001
Superoxide dismutase [Cu–Zn], SodC	Destroys radicals which are normally produced within the cells and which are toxic to biological systems	+ (p>0.05)	+ (p>0.05)	A0A5S9S8T8_9FLAO	P	Sod_Cu	Suzuki et al., 2001
KatB catalase	Serves to protect cells from the toxic effects of hydrogen peroxide	+	-	A0A5S9SXC2_9FLAO	P	Catalase,Catalase-rel	Suzuki et al., 2001
**TISSUE-DEGRADING ENZYMES**							
Chondroitin AC lyase, CslA	Chondroitinase-AC containing a C-terminal secretion signal family PL8_3	-	+	A0A5S9V218_9FLAO	N	DUF1573,Lyase_8,Lyase_8_C,Lyase_8_N	Pérez-Pascual et al., 2017
Sialidase, SiaA	Multimodular sialidase/Sialate O-acetylesterase/sialidase family GH33 containing a C-terminal secretion signal	-	+	A0A5S9REV9_9FLAO	G	BNR_2,BNR_assoc_N,CHB_HEX_C_1,Laminin_G_3	Pérez-Pascual et al., 2017

Sphingomyelinase, Sph	Endonuclease/Exonuclease/phosphatase family	-	+	A0A5S9TEZ1_9FLAO	S	Exo_endo_phos	Pérez-Pascual et al., 2017
Neutral ceramidase, Cer	Neutral alkaline nonlysosomal ceramidase	-	+	A0A5S9U028_9FLAO	S	Ceramidase_alk, Ceramidse_alk_C	Pérez-Pascual et al., 2017
Collagenase, Col	Hemolysin containing a C-terminal secretion signal. Cholesterol-dependent cytolysin	-	+	A0A5S9TI68_9FLAO	E	PKD,Peptidase_M43,fn3	Pérez-Pascual et al., 2017
Tenacilysin tly	Thiol-activated cytolysin	+ (0.06)	+ (1.11)	A0A5S9THM7_9FLAO	M	Thiol_cytolysin	Los et al., 2013
**TONB-DEPENDENT TRANSPORTERS**							
**Iron adquisition**	Hypothetical TonB-dependent outer membrane receptor	+ (1.84)	+ (0.25)	A05S9URL6_9FLAO	P	CarbopepD_reg_2,Plug,TonB_dep_Rec	
	Hypothetical TonB-dependent outer membrane receptor	+ (1.09)	+ (0.37)	A0A5S9TBY8_9FLAO			
	Hypothetical TonB-dependent outer membrane receptor	+ (0.85)	+ (0.27)	A0A5S9URM5_9FLAO			
	Hypothetical TonB-dependent outer membrane receptor	+ (1.72)	+ (0.25)	A0A5S9USH2_9FLAO			
	Hypothetical TonB-dependent outer membrane receptor	+	-	A0A5S9UVX0_9FLAO			
	Hypothetical TonB-dependent outer membrane receptor	+	-	A0A5S9RR19_9FLAO			
	Hypothetical TonB-dependent outer membrane receptor	+	-	A0A5S9TN86_9FLAO			
	Hypothetical TonB-dependent outer membrane receptor	+	-	A0A5S9S8J8_9FLAO			

	Hypothetical TonB-dependent outer membrane receptor	+	-	A0A5S9VXT0_9FLAO		
	Hypothetical TonB-dependent outer membrane receptor	+	-	A0A5S9SZ11_9FLAO		
	Hypothetical TonB-dependent outer membrane receptor	+	-	A0A5S9RLH1_9FLAO		
	Hypothetical TonB-dependent outer membrane receptor	+ (p>0.05)	+ (p>0.05)	A0A5S9SRR7_9FLAO		
	Hypothetical TonB-dependent outer membrane receptor	+ (p>0.05)	+ (p>0.05)	A0A5S9UMQ2_9FLAO		
	Hypothetical TonB-dependent outer membrane receptor	+ (p>0.05)	+ (p>0.05)	A0A5S9U999_9FLAO		
	Outer membrane receptor for ferrienterochelin and colicins	+ (p>0.05)	+ (p>0.05)	A0A5S9RJX3_9FLAO		
	Hypothetical TonB-dependent outer membrane receptor	+ (p>0.05)	+ (p>0.05)	A0A5S9SVU2_9FLAO		
	Hypothetical TonB-dependent outer membrane receptor	+ (p>0.05)	+ (p>0.05)	A0A5S9VD89_9FLAO		
	Hypothetical TonB-dependent outer membrane receptor	+ (p>0.05)	+ (p>0.05)	A0A5S9U4N9_9FLAO		
**TonB protein C-terminal, TonB**	Interacts with outer membrane receptor proteins that carry out high-affinity binding and energy dependent uptake into the periplasmic space of specific substrates. It could act to transduce energy from the cytoplasmic membrane to specific energy- requiring processes in the outer membrane, resulting in the release into the	+	-	A0A5S9TCC5_9FLAO A0A5S9TCA2_9FLAO A0A5S9UI67_9FLAO	M	TonB_C

	periplasm of ligands bound by these outer membrane proteins						
**Glycan harvesting**	SusC/RagA family TonB-dependent receptor	+ (1.06)	+ (0.15)	A0A5S9VVW7_9FLAO		H	CarbopepD_reg_2,Plug,TonB_dep_Rec
	SusC/RagA family TonB-dependent receptor	+ (1.89)	+ (0.16)	A0A5S9VF56_9FLAO		H	CarbopepD_reg_2,Plug,TonB_dep_Rec
	SusD/RagB family lipoprotein	+ (1.44)	+ (0.17)	A0A5S9VGZ0_9FLAO		H	SusD-like,SusD-like_2
	SusD/RagB family lipoprotein	+ (0.99)	+ (0.48)	A0A5S9VGS9_9FLAO		H	SusD-like,SusD-like_2
	SusC/RagA family TonB-dependent receptor	+	-	A0A5S9TK59_9FLAO<br>A0A5S9RJK5_9FLAO		H	CarbopepD_reg_2,Plug,STN,TonB_dep_Rec
**Iron regulated lipoproteins**	Imelysin family	+ (0.09)	+ (1.16)	A0A5S9U114_9FLAO<br>A0A5S9RU32_9FLAO<br>A0A5S9RVP5_9FLAO<br>A0A5S9RVE7_9FLAO		P	Peptidase_M75
**MEMBRANE BIOGENESIS**							
	Outer membrane protein OmpA family P60	+ (0.78)	+ (0.34)	A0A5S9U9Y8_9FLAO	M		OMP_b-brl,OmpA,TSP_3
	Extracellular matrix structural constituent	-	+	A0A5S9UTT1_9FLAO	M		CHU_C,DUF11

A

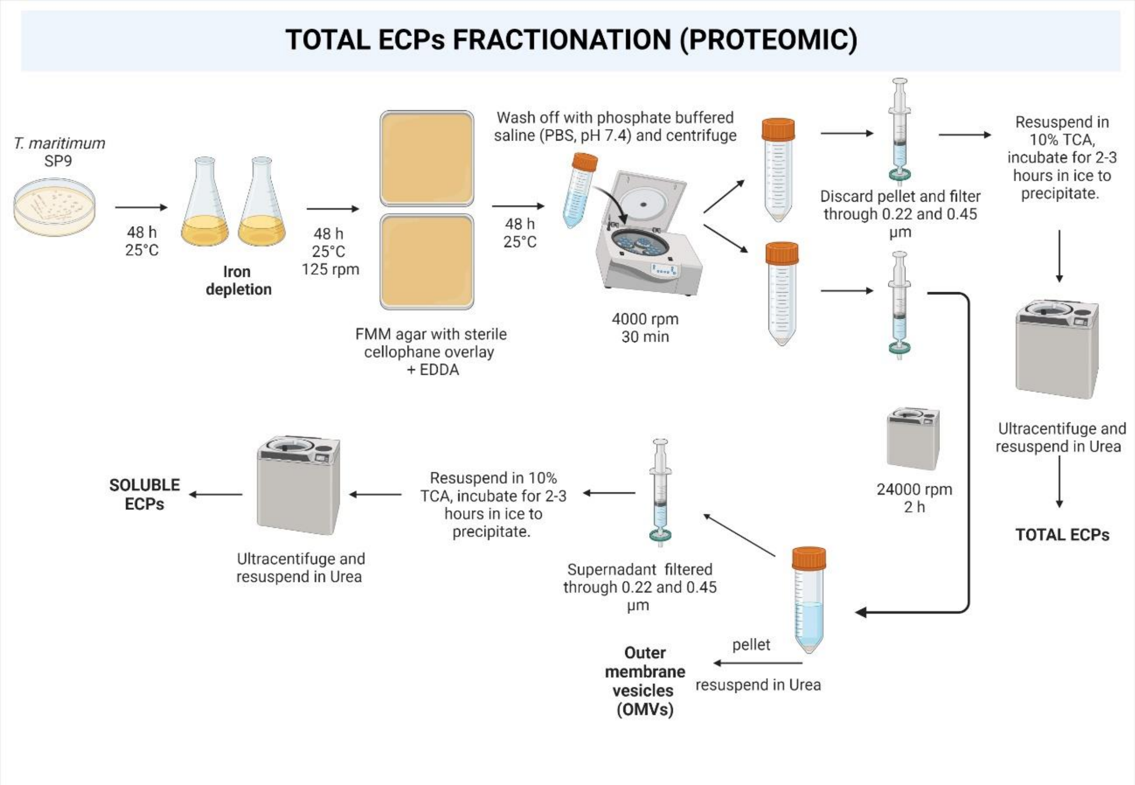

B

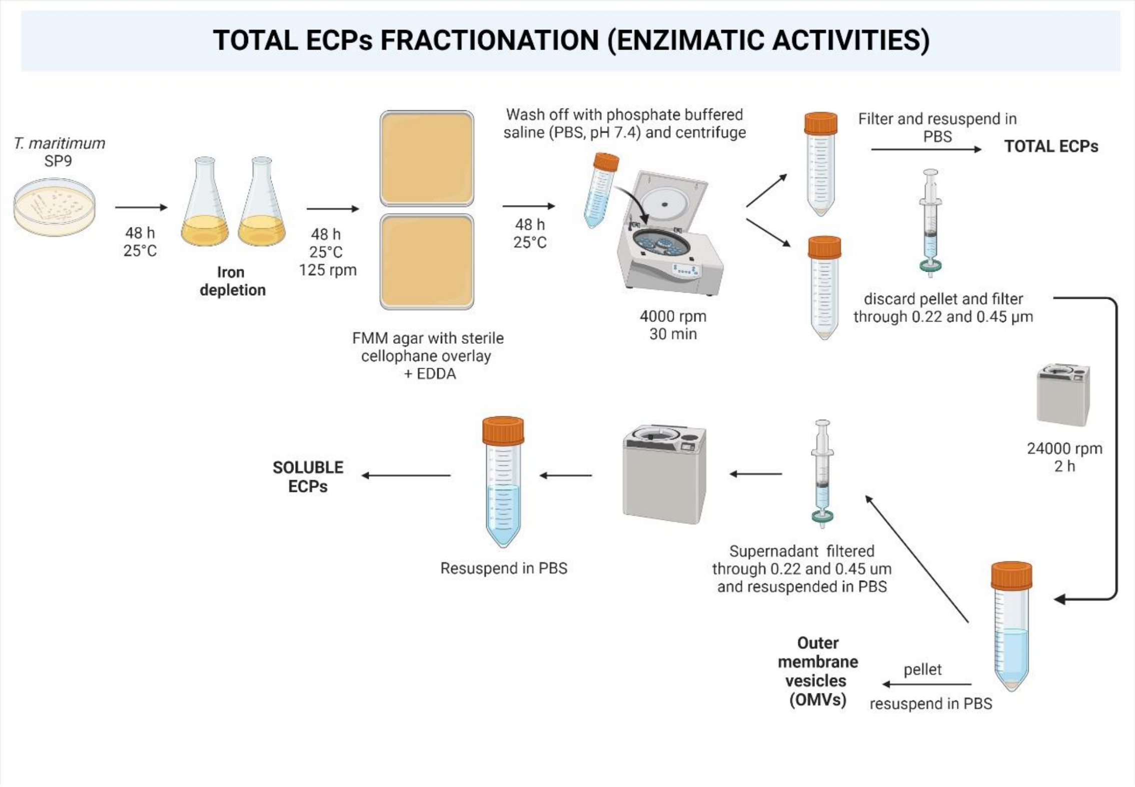

Figure S1. Preparation workflow. Total Extracellular products extraction (Total ECPs) and sub-cellular fractionation in outer membrane vesicles (OMVs) and Soluble extracellular products (SECPs) for proteomic analysis (A) and enzymatic activities assays (B).

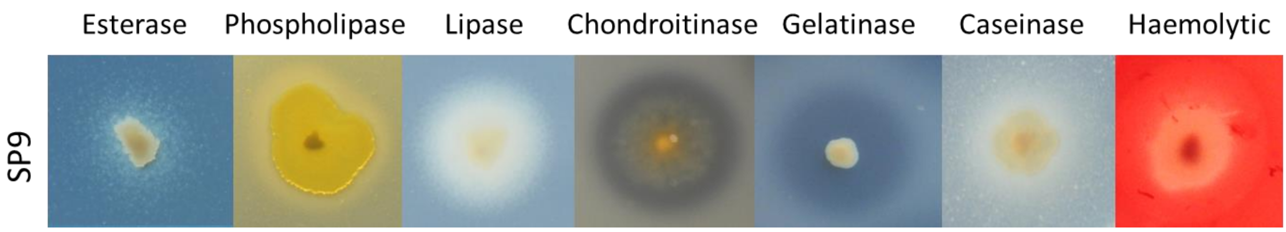

Figure S2. Protease, lipolytic and haemolytic activities of *Tenacibaculum maritimum* SP9 cells and outer membrane vesicles in FMM plates supplemented with different substrates.

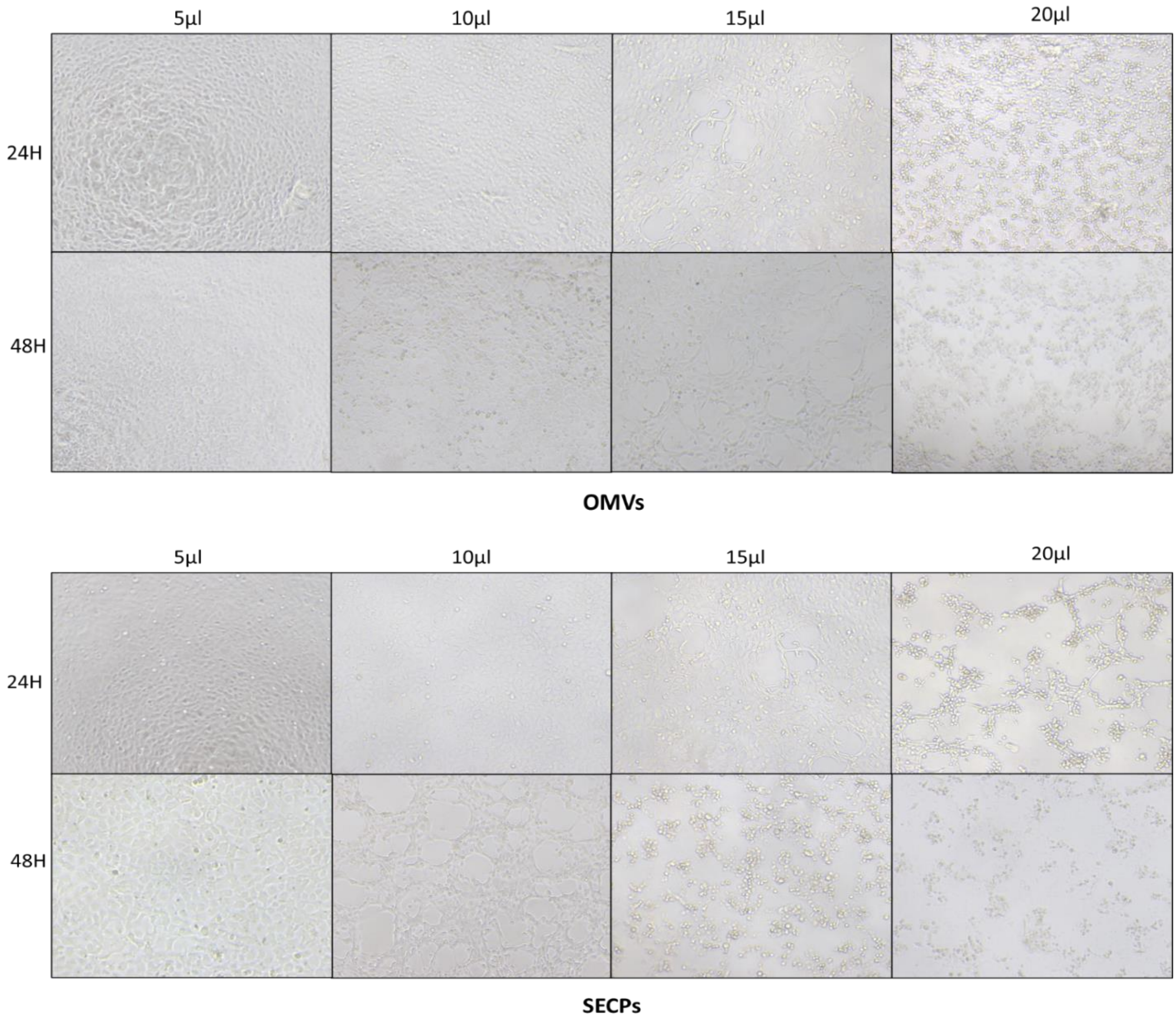

Figure S3. Cytotoxicity effect of OMVs and S-ECPs from *T. maritimum* in EPC cell lines. Magnification, 30X.
